# Supplementary material for: Frequency tunable magnetostatic wave filters with zero static power magnetic biasing circuitry
Source: Nat Commun. 2024 Apr 27;15:3582. doi: 10.1038/s41467-024-47822-3 (PMC11055899; doi:10.1038/s41467-024-47822-3)
Supplement: Supplementary file 1 — Supplementary Information [file 41467_2024_47822_MOESM1_ESM.pdf]

Frequency Tunable Magnetostatic Wave Filters With Zero Power Magnetic Biasing Circuitry

Supplementary Information

Xingyu Du[1], Mohamad Hossein Idjadi[1], Yixiao Ding[1], Tao Zhang[1], Alexander J. Geers[1], Shun Yao[1], Jun Beom Pyo[1], Firooz Aflatouni[1], Mark Allen[1], and Roy H. Olsson III[1*]

[1]Department of Electrical and Systems Engineering, University of Pennsylvania, Philadelphia, PA, USA
Email: rolsson@seas.upenn.edu

Table of Contents

Supplementary Note 1: Comparison of performance metrics for electrically continuous tunable bandpass filters 3

Supplementary Note 2: Overall Layout of the Magnetostatic Wave Resonators (MSWR) 5

Supplementary Note 3: Magnetic Probe Station Measurement Setup 6

Supplementary Note 4: Circuit Modeling for Magnetostatic Wave Resonators (MSWR) 7

Supplementary Note 5: Width Effect of the Aluminum Transducers 13

Supplementary Note 6: Length Effect of Magnetostatic Wave Resonators (MSWR) 16

Supplementary Note 7: Impedance Matching of the Magnetostatic Wave Filters (MSWF) 17

Supplementary Note 8: Frequency Tunability of Magnetostatic Wave Filters (MSWF) 19

Supplementary Note 9: Mode Analysis of Magnetostatic Wave Resonators (MSWR) 20

Supplementary Note 10: Circuit Modeling Procedure for Magnetostatic Wave Filters (MSWF) 25

Supplementary Note 11: Comparison of S_{12} and S_{21} 26

Supplementary Note 12: 1 dB Compression Measurement 27

Supplementary Note 13: Intermodulation Intercept Point (IIP3) Measurement 29

Supplementary Note 14: Magnetic Biasing Circuit Simulation for Magnetic Flux Density 35

Supplementary Note 15: Magnetic Feld Uniformity of the Magnetic Biasing Circuit 38

Supplementary Note 16: Magnetically Tunable Notch Filter 41

Reference 43

Supplementary Note 1: Comparison of performance metrics for electrically continuous tunable bandpass filters.

Type	Freq. Range (GHz)	Tuning Ratio	In-Band Insertion Loss (dB)	Out-of-band Rejection (dB)	Out-of-Band 3rd order input referred inter-modulation intercept point (OOB IIP3) (dBm)	Power Consumption	Size (cm^3)
This work (Planar YIG)	3.4-11.1	3.3:1	3.2-5.1	>25	>41	0	1.68
Planar YIG[1]	2-12	6:1	20-32	>40	Not reported	Electro-magnet (>58 W[2])*	Electro-magnet (75-99 [2])*
Planar YIG[3]	5.2-7.5	1.4:1	1.6-3	>20	Not reported	Electro-magnet (>58 W[2]) *	Electro-magnet (75-99 [2])*
Planar YIG[4]	11-16	1.5:1	~3.5	~60	Not reported	Electro-magnet (>58 W[2])*	Electro-magnet (75-99[2])*
YIG Sphere[5]	2-18	9:1	<6	>80	Not reported	Electro-magnet (2-10 W)	Electro-magnet (22.7)
Varactor[6]	1.6-2.1	1.3:1	2.4-3	>50	Not reported	Microwatts[7]	0.2
Varactor[8]	10.2-15.7	1.5:1	2.6-3.9	>35	Not reported	Microwatts[9]	0.4
Varactor[10]	8.4-9.2	1.1:1	3.5-5.7	~30	Not reported	Microwatts[11]	0.05
Varactor[12]	2.4-2.9	1.2:1	3.3-5.1	>10	Not reported	12 μW	0.05
RF MEMS[13]	2.8-5.2	1.8:1	0.1	>40	>60	0	~80
RF MEMS[14]	3.0-4.7	1.6:1	2-4	>50	Not reported	0	~8

Supplementary Table1. Performance metrics comparison for microwave tunable bandpass filters spanning from S band to X band (2-12 GHz). The indicated size encompasses the complete assembly of the filter, incorporating electromagnets for YIG-based filters.

*These papers reference the use of electromagnets to generate the external magnetic field required for the YIG material, yet they do not elaborate on the specific design of these electromagnets. However, in a related study conducted by Chen S. Tsai and Gang Qiu, details regarding the electromagnet dimensions and specifications are provided. The total electromagnet dimensions were approximately 63.5 mm (height) by 19.1 mm (width) by 61.8-81.4 mm (length), resulting in a volume range of 75-99 cm^3. The magnetic circuit employed two solenoids, each requiring a 1.2 A current and possessing a resistance of 20 Ω to generate a magnetic field of about 300 Gauss. Consequently, the power consumption for this electromagnet setup exceeded 58 W.

In Supplementary Figure 1, a size comparison in shown between this work and commercial YIG sphere-based tunable filters, namely the Omniyig Model C104F tunable filter operating from 4 to 8 GHz with electrical driver and the Omniyig Model M138 tunable filter operating from 2 to 8 GHz. The YIG sphere-based filters are notably larger due to the electromagnets required to adjust their resonance frequency.

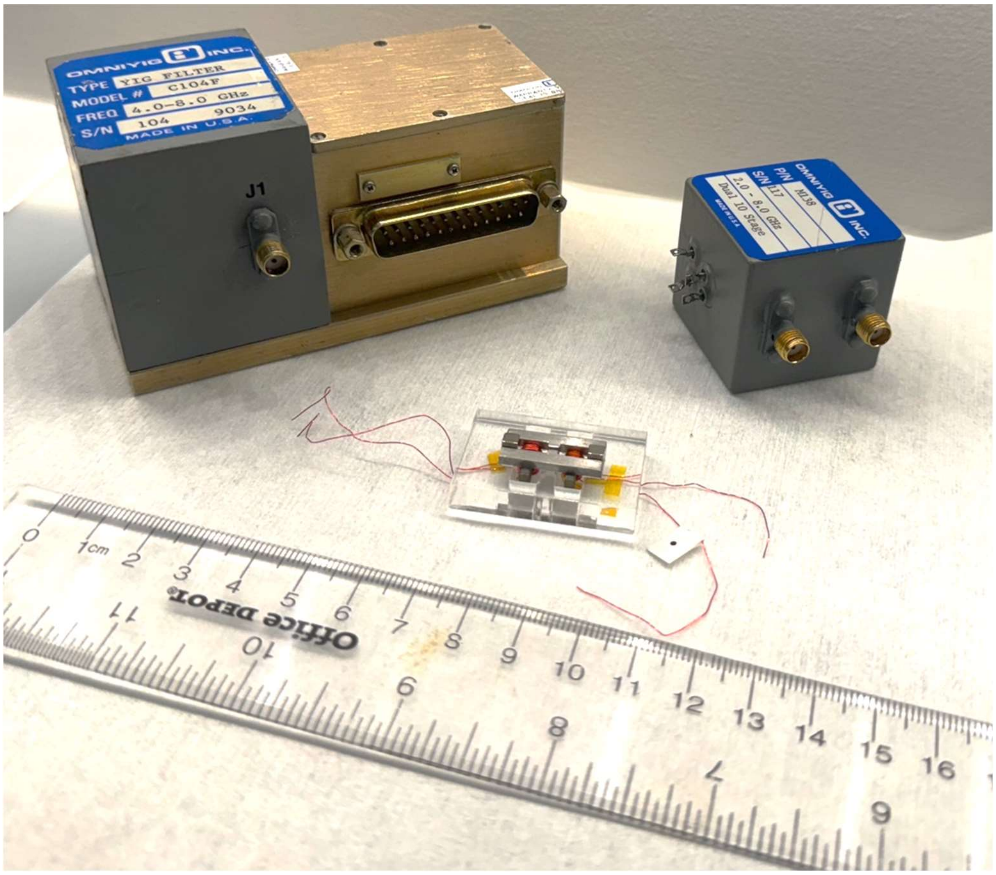

Supplementary Figure 1. A size comparison between this work and commercial YIG sphere-based tunable filters. Top left: Omniyig Model C104F tunable filter operating from 4 to 8 GHz with electrical driver. Top right: Omniyig Model M138 tunable filter operating from 2 to 8 GHz without the electrical driver. Bottom: tunable filter presented in this study.

Supplementary Note 2: Overall Layout of the Magnetostatic Wave Resonators (MSWR)

Supplementary Figure 2 depicts the arrangement of the MSSW devices. The measurement employed two pairs of Ground-Signal-Ground (GSG) probes with a pitch of 150 µm (GGB, 40A-GSG-150-P model). The ground pads possess a width of 130 µm, while the source pads have a width of 110 µm. To establish connectivity between the two ground pads, an Aluminum (Al) trace with a width of 60 µm is utilized. Beyond the signal contact pads, the Al traces undergo a reduction in width. This reduction achieves a minimum width of 5 µm in the Aluminum transducer nearest to the YIG regions, which are the coupling regions. The coupling efficiency with the magnetostatic wave is directly proportional to the current density. To address this, taper structures are incorporated to convert the 5 µm width of the coupling Al transducer to wider contact pads.

COMSOL simulations have been utilized to simulate the structure. In the absence of an external magnetic field, an isolation (S_{12}) of more than 25 dB can be achieved. The series resistance and inductance have been optimized by employing an aluminum thickness of 2 µm. The series components and isolation characteristics are further analyzed in the subsequent sections.

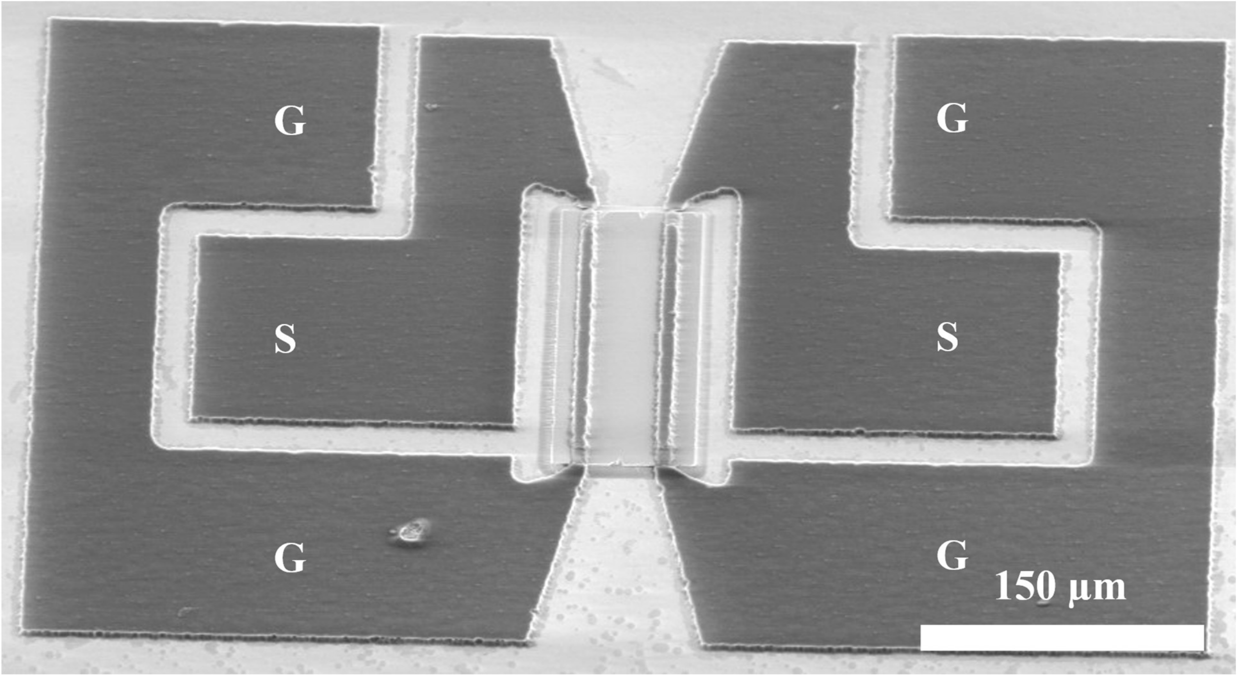

Supplementary Figure 2. SEM image of MSWF with the transducers on top of the YIG setup with width of 150 µm and length of 70 µm. Ground (G) and Signal (S) contact pads are shown in the image.

Supplementary Note 3: Magnetic Probe Station Measurement Setup

Supplementary Figure 3 illustrates the experimental configuration utilized for measuring the MSSW filters. An array of MSSW filters were fabricated on a YIG/GGG (Yttrium Iron Garnet/Gadolinium Gallium Garnet) substrate and positioned on a 5 mm thick acrylic spacer. The Acrylic spacer was centrally placed on a metallic stage. To ensure a consistent and adjustable magnetic field across the MSSW filters, two electromagnets were positioned on either side of the metallic stage. The magnitude of the magnetic field was regulated by controlling the current within the coils of the electromagnets, employing closed loop sensor feedback control for precise adjustments.

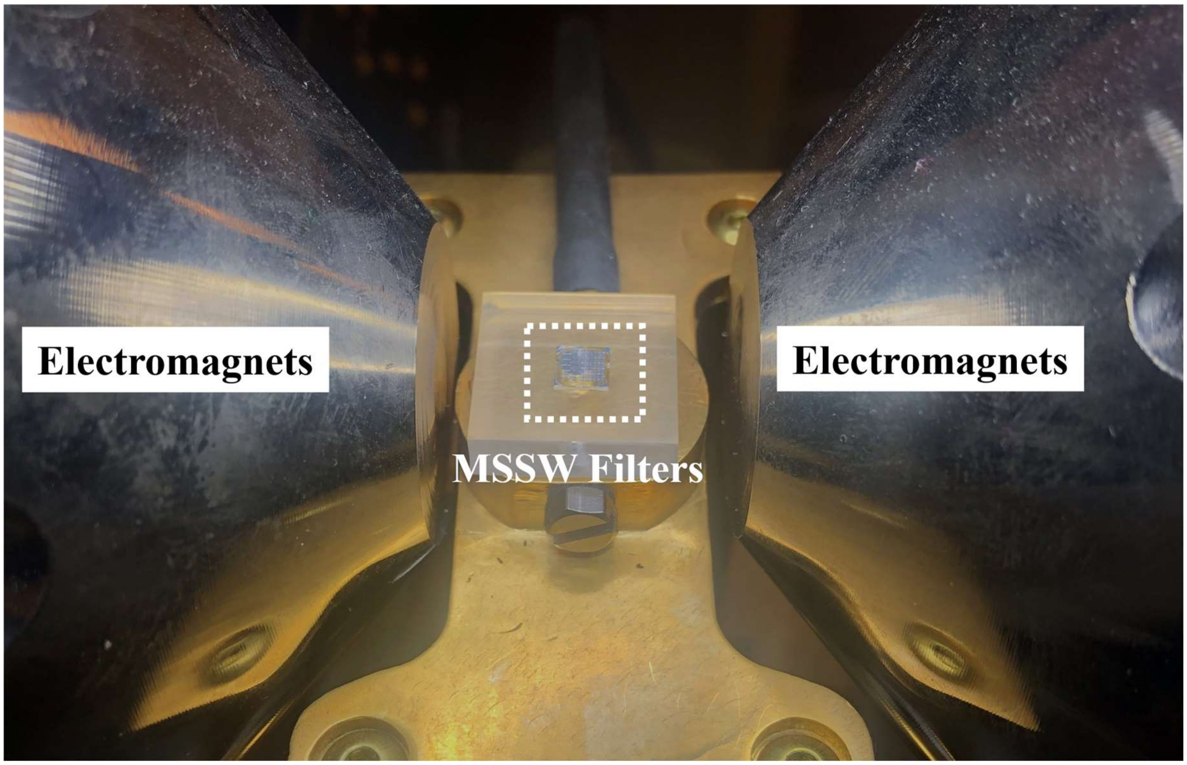

Supplementary Figure 3. MSSW filters inside the magnetic probe station measurement setup.

Supplementary Note 4: Circuit Modeling for Magnetostatic Wave Resonators (MSWR)

Following the fabrication and measurement of the two-port MSWF, using the magnetic probe station, the performance of the fabricated MSWR and MSWF were evaluated by studying the circuit model of the devices. In the measurement of the MSWF, the full S-parameters of the two ports were recorded. To determine the characteristics of the one-port MSWR device, we utilized the values obtained from either Z_{11} or Z_{22} of the MSWR, representing the impedance when the other port is open.

As outlined in Supplementary Note 6, the MSSW filters exhibit multiple modes owing to the highly dispersive nature of spin waves in a planar rectangular YIG cavity. Consequently, these different modes are closely clustered together in frequency. Unlike spurious-free single mode resonators, the frequency response of MSSW filters can display numerous local maxima and minima. The anticipated behavior of an ideal resonance, characterized by maximum impedance at the resonance frequency (f_s) and minimum at anti-resonance frequency (f_p), becomes challenging to observe. Consequently, the accurate extraction of circuit parameters for MSWR becomes a laborious task. This issue is less pronounced in devices with low coupling (K^2), primarily because the narrow frequency spacing between f_s and f_p results in fewer spurious modes within that range.

In a conventional acoustic resonator, a multi-mode Modified Butterworth-Van Dyke (MBVD) circuit can be established by introducing several parallel branches of the motional resistor R_x, the motional inductor L_x, and the motional capacitor C_x to the main mode resonance tank. The circuit parameters derived from these additional parallel branches enable the calculation of the coupling coefficient and quality factor of the spurious mode.[15] Inspired by the multi-mode MBVD circuit, Figure 2 illustrates the circuit diagram of MSWR with a total of p modes. In each resonance tank (designated as the nth tank in the circuit), there is a parallel magnetostatic resistor R_m, one magnetostatic inductance L_m,and one magnetostatic capacitor C_m. These resonance tanks are connected in series, the impedance of the resonance tank in the MSWR reaches its maximum at resonance and returns to a minimum at frequencies away from the resonance. This behavior contrasts with traditional acoustic resonators, where additional branches would remain open when only the main branch is resonating. In MSSW resonators, the additional branches remain electrically shorted when only the main branch is resonating. This unique property of MSWR leads to the S_{11} parameter of both the MSWR and MSWF exhibiting a bandstop characteristic, indicating the Z_{11}impedance reaches the maximum at resonance frequency. Additionally, the S_{12} parameter of the MSWF shows a bandpass characteristic, indicating the Z_{12}impedance reaches the minimum at resonance frequency.

As for a single mode MSWR, the impedance of a resonator is the series combination of series resistor R_s and series inductance L_s and the resonance tank. It can be expressed as follows.

$$Z_{single} = Z_{series} + Z_{tank\_single} = R_s + j\omega L_s + \frac{1}{\frac{1}{R_m} + \frac{1}{j\omega L_m} + j\omega C_m} \tag{S1}$$

The total impedance of an MSWR's tank consisting of a total of p modes can be expressed by combining the impedance of each individual resonance tank, denoted as: Z_{tan} , Z_{tank2} , Z_{tank3}, …… , Z_{tankp}. Consequently, the total impedance of a MSWR, Z_{total}, can be described as follows:

$$Z_{total} = Z_{series} + \sum_{n=1}^{p} Z_{tank\_n} = R_s + j\omega L_s + \sum_{n=1}^{p} \frac{1}{\frac{1}{R_{m1n}} + \frac{1}{j\omega L_{m1n}} + j\omega C_{m1n}} \quad \text{(S2)}$$

With the establishment of the multi-mode circuit model for MSW devices, the process flow employed to extract the circuit parameters is described in Supplementary Fig. 4. The determination of these parameters assumes a critical role in precisely characterizing the device's behavior and facilitating performance optimization.

The procedure commences by importing the measured impedance data, Z_{mea}. Subsequently, the R_s and L_s are calculated from the imaginary and real parts of the off-resonance impedance, respectively. When the frequency significantly deviates from the resonance, the overall Z_{11} impedance of MSWR can be expressed as, Z_{series}.

$$Z_{series} = R_{s1} + j\omega L_{s1} \quad \text{(S3)}$$

Subsequently, the impedance response is analyzed to identify the frequency peaks of interest. These peaks, usually corresponding to local maxima with the highest magnitude, are then ranked based on their impedance values. For instance, the first peak, representing the resonance with the largest impedance, is used to calculate the circuit parameters of the resonance tank associated with the largest. The second peak represents the second-largest resonance impedance, and so on. By examining the resonance peaks in descending order, it becomes possible to observe and analyze the impedance changes of smaller resonance tanks, as the significant impedance of larger resonance modes can conceal the impedance response of smaller modes. To accurately determine the fitting frequency range, a local minimum preceding the selected peak and a local minimum following it are identified. This frequency range is crucial as it captures the dominant influence of the corresponding resonance tank's impedance on the overall circuit behavior. By focusing on this range, a precise characterization of the specific resonance mode can be achieved.

After that, the initial guess parameter for the resonance tank circuit parameters: R_m, L_m, and C_m can been estimated for the initialization of the multi-mode circuit model recursive fitting. The initial condition for R_m is the frequency response's maximum magnitude of the impedance. The Q can be approximated as the ratio of f_s over this peak's 3-dB bandwidth. The initial condition for L_m and C_m can be expressed as:

$$L_m = \frac{R_m}{2\pi f_s Q} \quad \text{(S4)}$$

$$C_m = \frac{1}{L_m (2\pi f_s)^2} \quad \text{(S5)}$$

After obtaining the initial values for R_m, L_m, and C_m, the random search process is employed to explore the parameter space for these three circuit parameters. This process begins by generating a series of three sets of random numbers that deviate from the initial three parameters. These random numbers serve as the test parameters for the subsequent analysis. By incorporating these test parameters into the circuit model, the corresponding impedance response of Z_{fit} can be

computed as the combination of the impedance of the series components with the impedance of the previously fit resonance tank, if any, and the resonance tank of interest with random generated test parameters.

In order to compare between the calculated impedance response and the measured impedance data, an error function of $\sigma(Z_{fit}, Z_{mea})$ can be calculated from the magnitude of the fit data, $dB(Z_{fit})$ and the measured data, $dB(Z_{mea})$ and the phase of the fit data, $phase(Z_{fit})$ and the measured data, $phase(Z_{mea})$, which is defined as:

$$\begin{aligned}\sigma(Z_{fit}, Z_{mea}) &= \sigma_{dB} + \sigma_{phase} \\ &= \left|\mathrm{Norm}(\mathrm{dB}(Z_{fit})) - \mathrm{Norm}(\mathrm{dB}(Z_{mea}))\right| \\ &+ \left|\mathrm{Norm}(\mathrm{phase}(Z_{fit})) - \mathrm{Norm}(\mathrm{phase}(Z_{mea}))\right|\end{aligned} \tag{S6}$$

The normalization function, denoted as $Norm(x)$, serves to eliminate the absolute scale difference between phase and magnitude. It can be mathematically expressed as follows:

$$Norm(x) = \frac{x - \mathrm{mean}(x)}{\mathrm{std}(x)} \tag{S7}$$

In this equation, x is the data of interest. The $mean(x)$ and $std(x)$ are the average and standard deviation of the data.

The error function is utilized to evaluate all the test parameters generated during the random search process. The test parameters associated with the smallest error function value are saved, as they indicate a closer match between the modeled impedance response and the measured data. This process of random number generation and error function evaluation is repeated multiple times until the error function converges, indicating a satisfactory level of parameter optimization. Once the error convergence is achieved, the circuit extraction process concludes with key fitting results for R_m, L_m, and C_m. These results represent the optimized circuit parameters that best align with the measured impedance data. Subsequently, the recursive fitting procedure moves on to the next peak of interest and performs the fitting for the resonance tank associated with the next mode. This iterative approach ensures comprehensive characterization of each resonance mode and the determination of their respective circuit parameters.

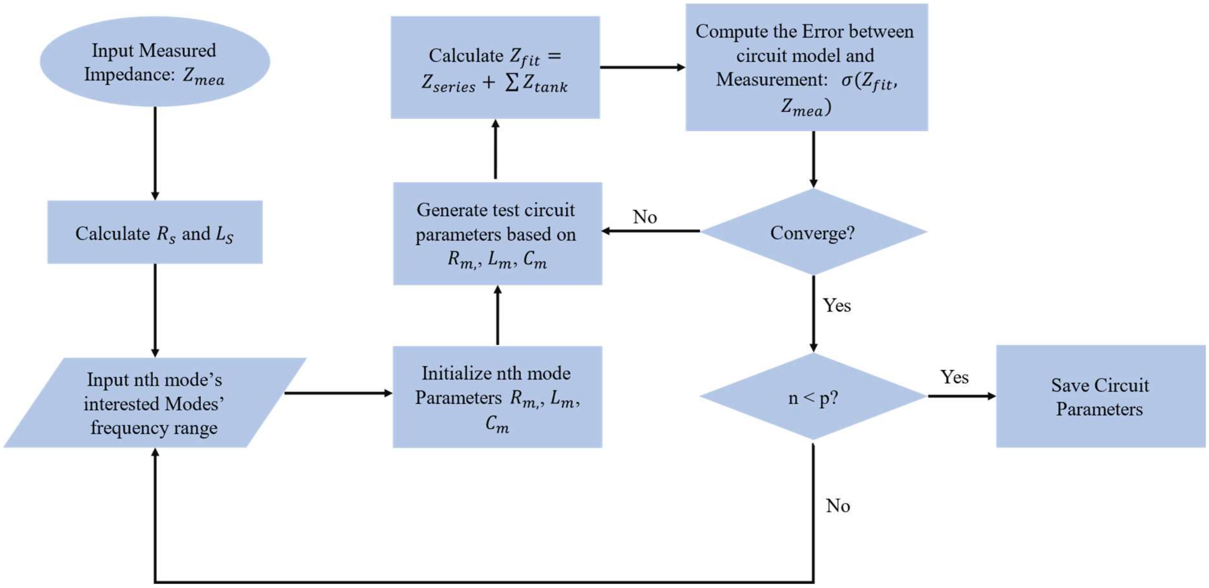

Supplementary Figure 4. Multi-resonance recursive fitting scheme for accurate extraction of the MSWR model with multiple resonance models.

In Figure 2, a comparison of the multi-mode circuit model, single-mode circuit model, and measurement are presented. Supplementary Figure 5 illustrates the details of each resonance tank in the multi-mode circuit model. In this MSWR with width of 200 μm and length of 70 μm, the top five local maximum are selected and the multi-mode recursive fitting was performed with five resonance tanks. By systematically generating and evaluating test parameters, this approach successfully identifies the parameter values that result in the closest agreement between circuit model and the measured data. The series R_s is 0.88 Ω and the series L_s is 0.3436 nH. The circuit parameters for the resonance tanks are shown in Supplementary Table 2.

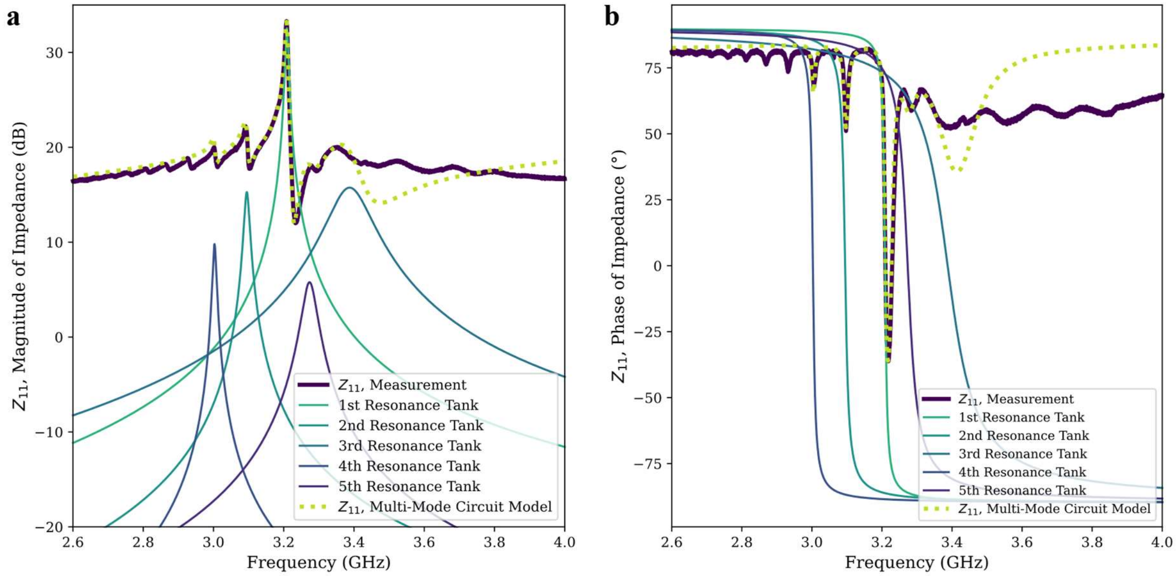

Supplementary Figure 5. Example of the multi-resonance recursive fitting using measurement of typical MSWR with width of 200 μm and length of 70 μm with transducer width of 4 μm. (a) Magnitude comparison between the measurement and the circuit model. (b) Phase comparison between the measurement and the circuit model.

	Resonance Tank #1	Resonance Tank #2	Resonance Tank #3	Resonance Tank #4	Resonance Tank #5
Resonance Frequency (GHz)	3.2	3.1	3.3	3.0	3.3
R_m (Ω)	42	5.8	6.1	3.1	1.9
L_m (pH)	5.8	1.4	9.7	0.56	1.2
C_m (nF)	0.4	1.9	0.23	5.0	2.0

Supplementary Table 2. Summary of the circuit parameters for multi-mode circuit model of MSWR with W = 200 μm, L = 70 μm, and transducer width of 4 μm.

Another application for the circuit model is to understand the effect of series resistance and inductance, as the spin wave Q-factor can be calculated from the circuit model. The spin wave Q-factor is defined as:

$$Spin\ Wave\ Q = \frac{R_m}{2\pi L_m} \tag{S8}$$

Supplementary Figure 6 illustrates the spin wave Q-factor of various MSWR with different widths. The spin wave Q-factor and device Q-factor follow similar trends with frequency. The maximum spin wave Q-factor of 1777 for W = 150 µm, L = 70 µm at 11.5 GHz can be achieved. Compared to device Q-factor, spin wave Q-factor removes the impact of the Al trace resistance on overall device performance and is about 30 % ~ 50% higher than the device Q. This indicates significant energy is dissipated through the series resistance.

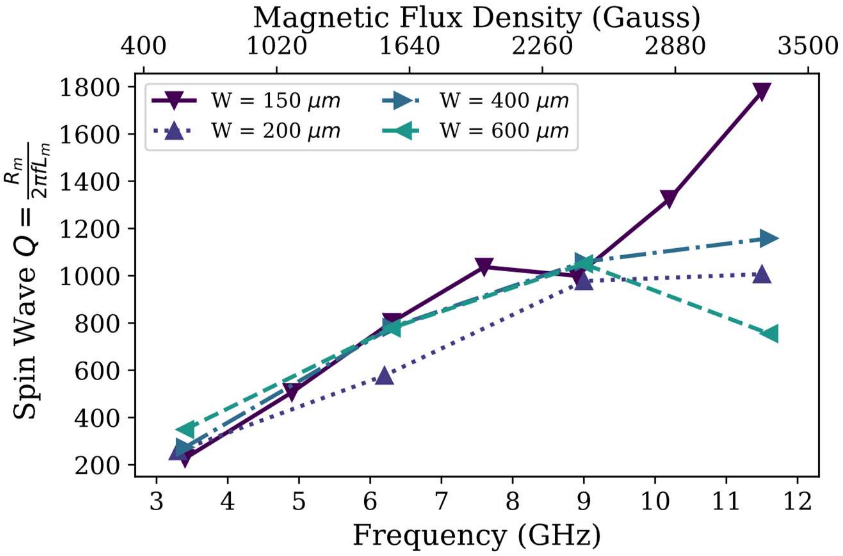

Supplementary Figure 6. Comparison of MSWR spin wave Q-factor for different width of YIG cavity.

Supplementary Note 5: Width Effect of the Aluminum Transducers

In order to achieve a low insertion over a broad frequency tuning range, the effect of the width of the aluminum (Al) transducers has been studied. A 2-dimensional finite element simulation was performed in COMSOL [16]to simulate the total integration of the magnetic flux density in the YIG when a DC current is applied to the aluminum trace line. The aluminum transducer was simulated with a trapezoidal shape to better represent the wet etching profile. The change in series resistance of the aluminum transducers with Al width is much less than 1 Ω when the width of the aluminum transducer decreases from 19 µm to 4 µm. Thus, a constant current assumption of 1 A was used for the simulation. Supplementary Figure 7 shows that a narrower Al transducer achieves higher magnetic flux, which is mainly due to the increase of the y component of the magnetic flux.

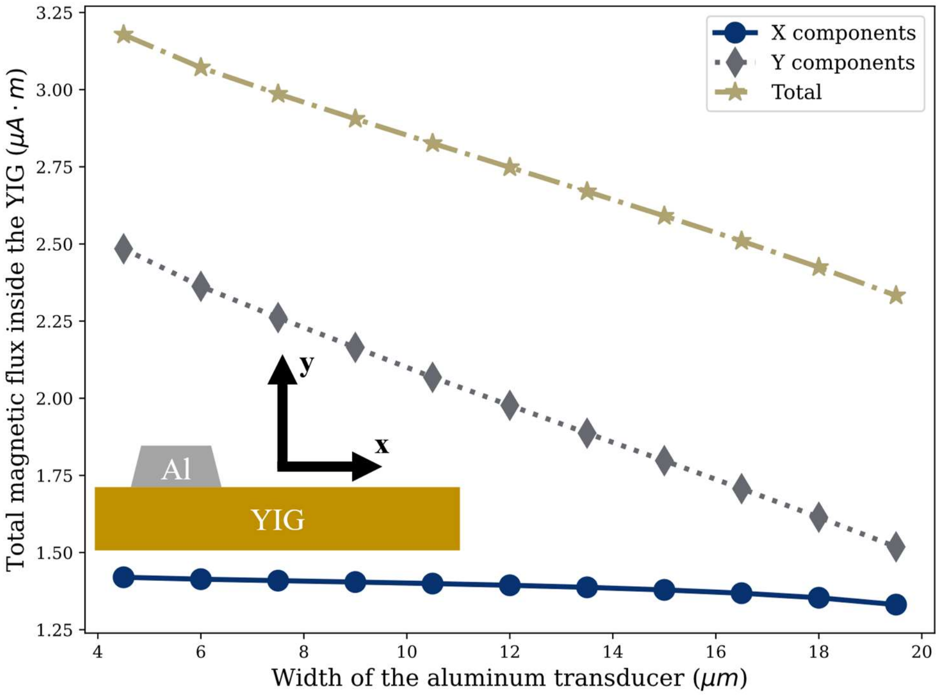

Supplementary Figure 7. COMSOL simulation results on the magnetic flux inside the YIG with respect to the width of the aluminum transducer.

As a result of the increased magnetic field inside the YIG cavity, the R_m and insertion significantly improve between 3.4 ~ 12.9 GHz, as shown in Supplementary Figure 8. The increased FOM does not translate into an improvement in insertion loss for frequencies above 12.9 GHz because the MSWR resonator is over coupled to the 50 Ω source impedance.

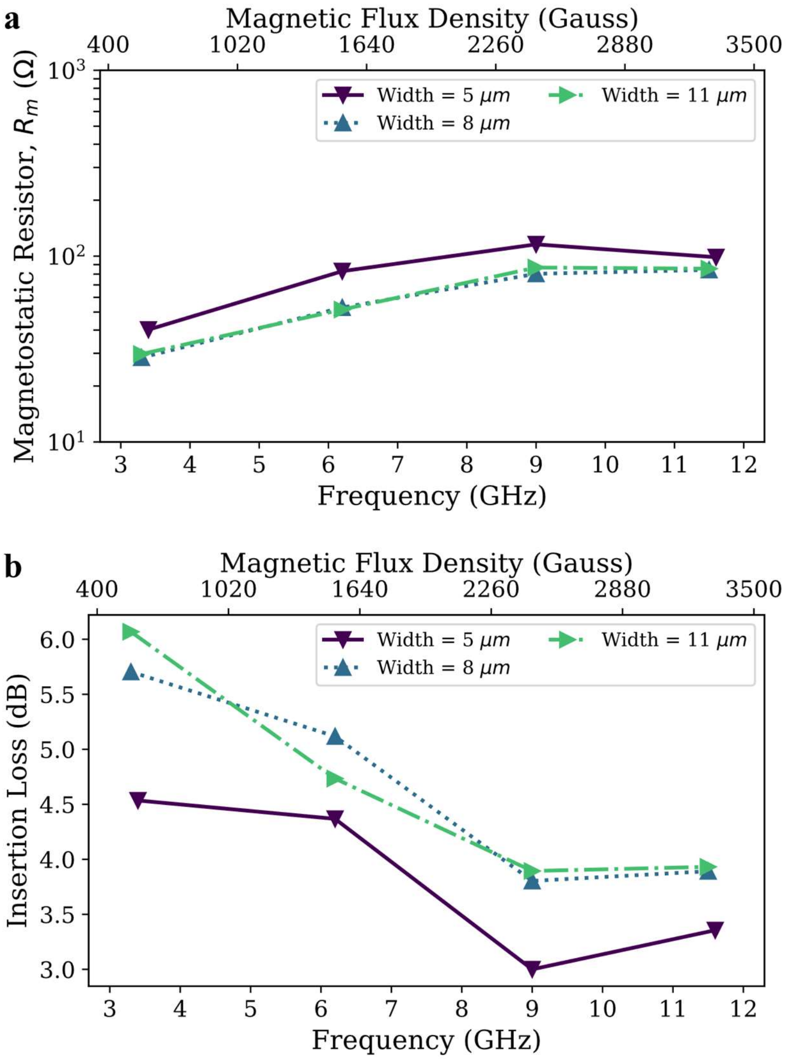

Supplementary Figure 8. Effect of the aluminum transducer width on the (a) radiation impedance and (b) insertion loss. The MSWF is designed with constant W = 200 µm and L = 70 µm.

Supplementary Figure 9 shows a typical frequency response for different Al transducer widths. A narrower aluminum transducer slightly reduces the out-of-band attenuation of the tunable filters as the increase of the current density in the aluminum trace slightly enhances the direct inductive coupling between the two ports.

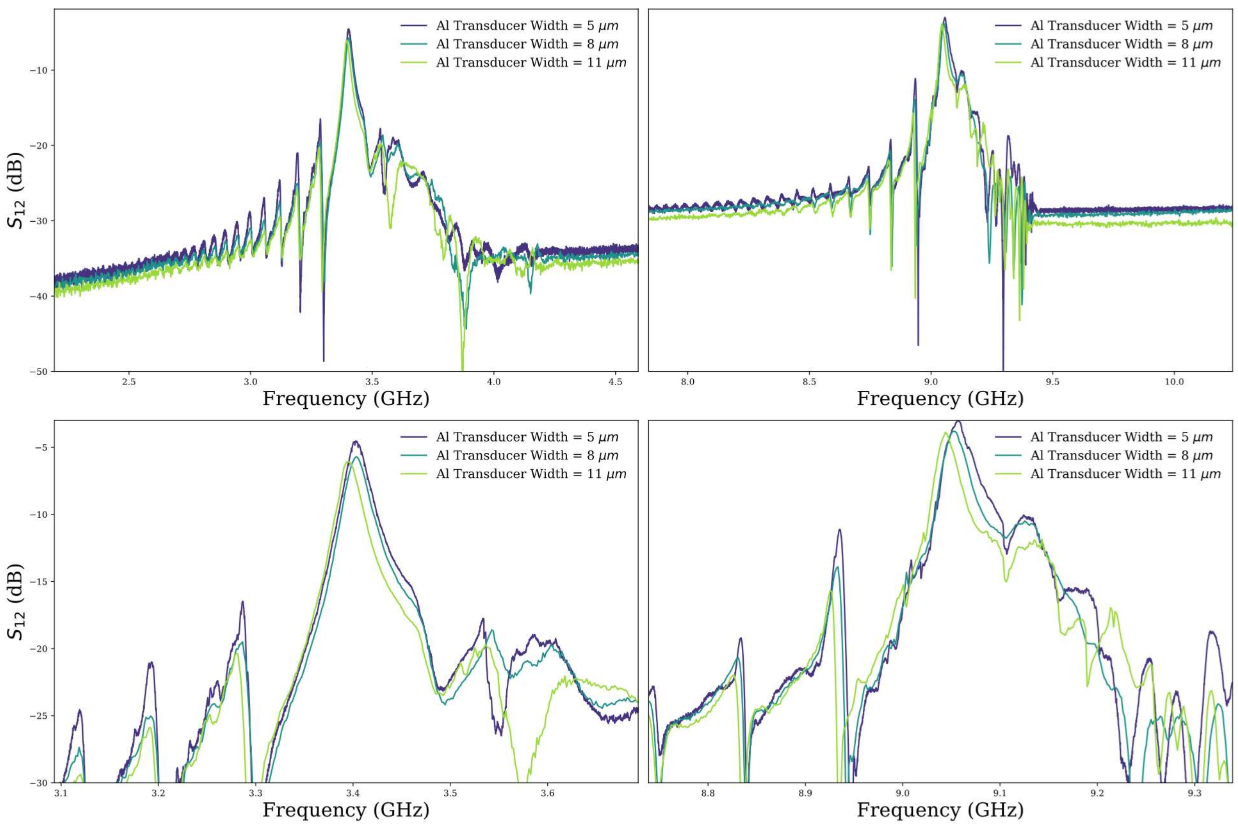

Supplementary Figure 9. Typical frequency responses of different MSSW filters with Al transducer widths of 5 μm, 8 μm, and 11 μm at a magnetic field of 554 and 2405 Gauss. The MSWF is designed with constant W = 200 μm and L = 70 μm.

Supplementary Note 6: Length Effect of Magnetostatic Wave Resonators (MSWR)

Supplementary Figure 10 compares the effect of length on MSWR K^2, Q-factor, FoM, and magnetostatic resistance R_m.

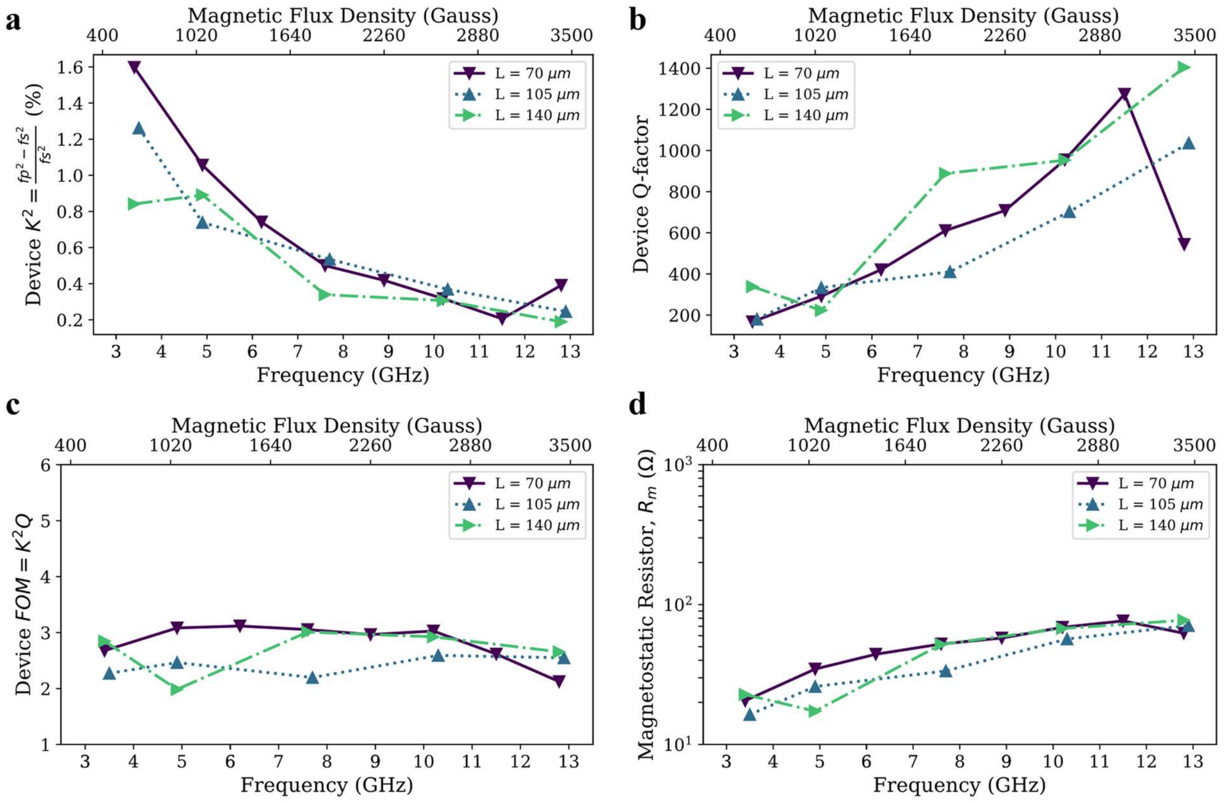

Supplementary Figure 10. Comparison of MSWR for various YIG cavity lengths with a constant width of 150 µm. (a) The effect of YIG cavity length on the device coupling coefficient, (b) The effect of YIG cavity length on the device Q-factor, (c) The effect of YIG cavity length on the device FOM, (d) The effect of YIG cavity length on the magnetostatic resistance, R_m.

Supplementary Note 7: Impedance Matching of the Magnetostatic Wave Filters (MSWF)

Figure 3 (d) illustrates that R_m increases with the external magnetic field. For MSWR with W = 150 μm, R_m is greater than 50 Ω for frequencies above 7.6 GHz and increases to 77 Ω at the frequency of 11.5 GHz. For the MSWR with W = 600 μm, the R_m is greater than 50 Ω for all frequencies with a minimum of 115 Ω at 3.4 GHz and the maximum of 363 Ω at 11.6 GHz. This difference in R_m causes different degrees of impedance mismatch for the MSWF vs. frequency.

Supplementary Figure 11 shows the return loss of the MSWF at the frequency where the S_{12} reaches its peak. Due to the increase of the magnetostatic resistance of the MSWF with W = 600 μm, this return loss decreases from 18 dB and 22 dB at 3.4 and 6.3 GHz, respectively, to 14 dB and 8 dB at 9 GHz and 11.6 GHz, respectively. As a contrast, the MSWF with W = 150 μm shows a minimum return loss of 6 dB at 3.4 GHz and achieves its maximum of 13 dB at 10.2 GHz. For the MSWF with W = 150 μm or W = 200 μm, the maximum of S_{11} is at the same frequency with the maximum of S_{12}, indicating the same fundamental mode. However, the large maximum R_m of the devices with wider YIG cavities caused the maximum return loss to occur at a higher order mode of the MSWF where the R_m of that higher order mode is closer to the 50 Ω termination impedance. At the fundamental mode where the maximum impedance is achieved, most of the signal is reflected back to the source and thus the insertion loss of the fundamental mode is high.

Supplementary Figure 12 depicts the insertion loss of the MSWF when terminated with 50 Ω. Due to the difference in the degree of the impedance matching, the insertion loss of the filters with a narrower YIG cavity (W = 150 or 200 μm) are more consistent across the frequency band whereas the insertion loss of the wide YIG cavity (W = 400 or 600 μm) decreases with increasing frequency.

To reduce the loss of the W = 600 μm devices across the filter band, an external series inductor and parallel capacitor can be used to achieve better matching to the 50 Ω termination impedance, but this requires matching networks that are tunable with frequency. Therefore, for the purpose of achieving a broad frequency tuning range of the filters, the MSWR should not only achieve the maximum FOM but also maintain good impedance matching to the 50 Ω source impedance across the tunable frequency range.

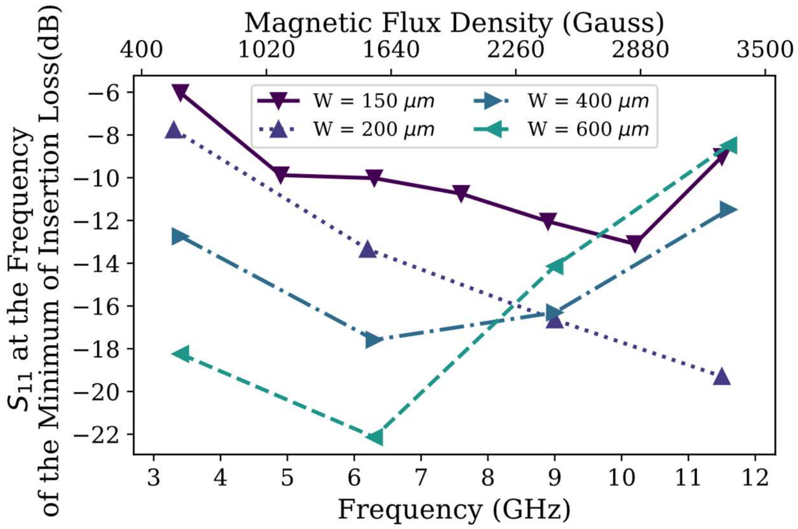

Supplementary Figure 11. S_{11} where S_{12} reaches the maximum vs. frequency.

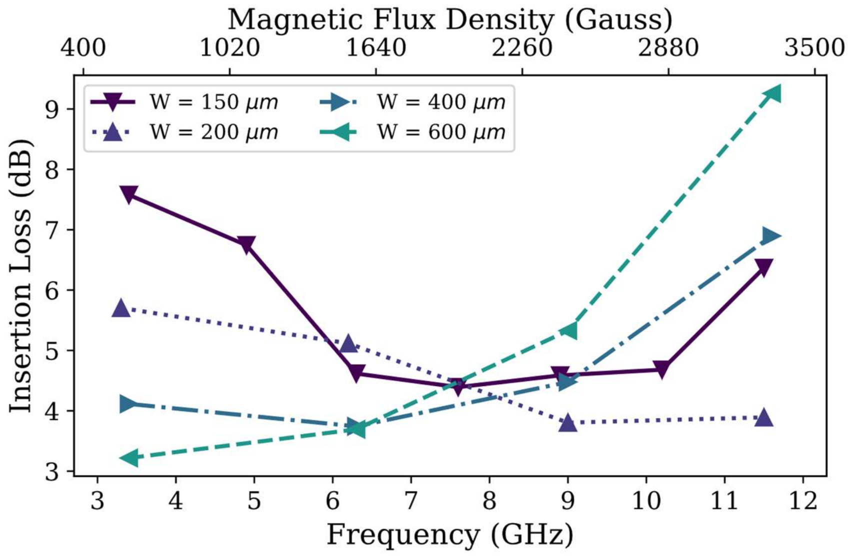

Supplementary Figure 12. Insertion loss vs. frequency. All the devices are made with aluminum transducers with width of 7 μm.

Supplementary Note 8: Frequency Tunability of Magnetostatic Wave Filters (MSWF)

Supplementary Figure 13 illustrates the tunability of the MSSW filters via applied magnetic field. In all the four different devices, the relationship of the main resonance frequency with respect to the applied magnetics bias field is linear with a slope of 2.9 MHz/Gauss. Here the resonance frequency is defined as the frequency where the loss reaches its minimum. This tunability is similar to devices reported in previous studies. [17, 18]

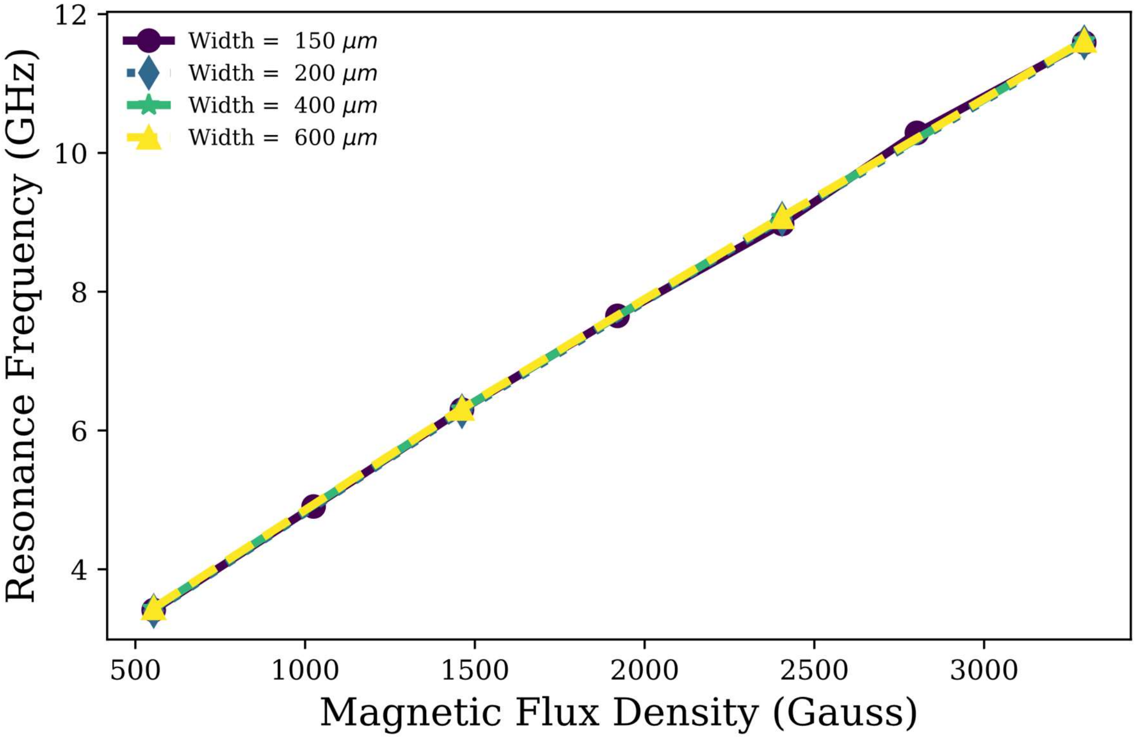

Supplementary Figure 13. Tunability of MSSW resonators. resonance frequency vs. applied magnetic field.

Supplementary Note 9: Mode Analysis of Magnetostatic Wave Resonators (MSWR)

Because the MSSW travels along the surface of the YIG film and is reflected to the other side of the YIG surface at the straight edges, the resonant conditions are when the following equation is met:[19]

$$2k_y L = 2\pi n \qquad n = 1,2,3, \ldots.. \tag{S8}$$

Where k_y is the average wavenumber for the top and bottom surfaces. As the length increases, the spacing between the resonance modes decreases in wavenumber, due to the strong dispersion of the MSSW. Although a wider MSWR can provide higher FoM and lower insertion loss, it also contains additional spurious responses akin to the response of a multimode waveguide associated with the width of the YIG structure. The dispersion relation with the width modes can be expressed as: [20]

$$exp(2Md) = \frac{\Omega_m M + \Omega_k + (\Omega_H^2 - \Omega^2)(M - N)}{\Omega_H M - \Omega_k + (\Omega_H^2 - \Omega^2)(M + N)} \times \frac{\Omega_m M - \Omega_k + (\Omega_H^2 - \Omega^2)(M - N\tanh(Nt)}{\Omega_m M + \Omega_k + (\Omega_H^2 - \Omega^2)(M + N\tanh(Nt)} \tag{S9}$$

Inside the film, we have

$$M^2 = \mathrm{k}_x^2 = \frac{(\frac{n\pi}{w})^2}{\mu_1} + k_y^2 \tag{S10}$$

And outside

$$N^2 = \mathrm{k}_x^2 = (\frac{n\pi}{w})^2 + k_y^2 \tag{S11}$$

where $\mu_1 = 1 - \Omega_H/(\Omega^2 - \Omega_H^2)$, $\Omega = \omega/\nu 4\pi M_s$, $\Omega_H = H/4\pi M_s$, ω is the angular frequency, ν is the gyromagnetic ratio of 2.8 MHz/Oe for YIG, and 4π Ms is the saturation magnetization of the YIG film which is 1780 Gauss, H is the external magnetic field applied, t is the distance between the YIG film and the ground plane which can be chosen as any arbitrary large number here.

The calculated results are shown in Supplementary Figure 14 and the measurements of the S_{12} frequency response for the MSWFs with widths of 200 µm, 400 µm, and 600 µm are shown in Supplementary Figure 15. Because the width of the MSWF is much smaller than the electromagnetic wavelength at these frequencies and the current remains almost constant along the transducer, only odd order width modes can be excited. The frequency spacing between two adjacent width modes increases for narrower YIG cavities. The frequency response of W = 600 µm and W = 150 µm confirms this result. There is also a frequency shift for the main resonance mode with increases in width, as can be seen from the dispersion curve where a constant wavenumber corresponds to a higher frequency as the width increases. As both theoretical calculation and

experimental measurements confirm, the MSSW becomes more dispersive at higher frequency for wider MSSW filters.

Supplementary Figure 16 and 17 illustrate the S_{11} frequency response of devices with varying widths and lengths. Consistent with the earlier findings, it can be observed that longer and wider MSWFs exhibit more spurious responses. Additionally, wider MSWFs display a lower minimum value of S_{11}. However, the length of the MSWF does not significantly affect the minimum value of S_{11}, as the increase in R_m is primarily associated with the width rather than the length. These observations further support the conclusions regarding the influence of width and length on the spurious responses and impedance characteristics of the MSWRs.

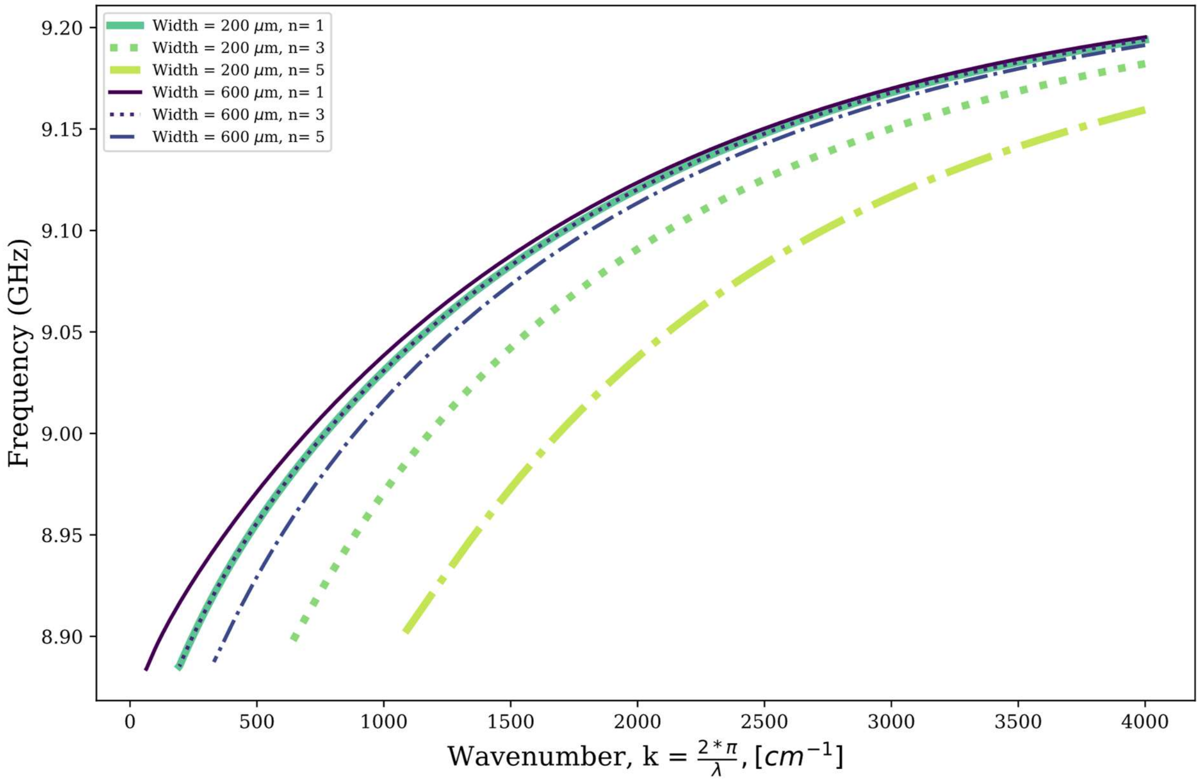

Supplementary Figure 14. Dispersion curves for MSWF width modes with magnetic flux density of 2405 Gauss.

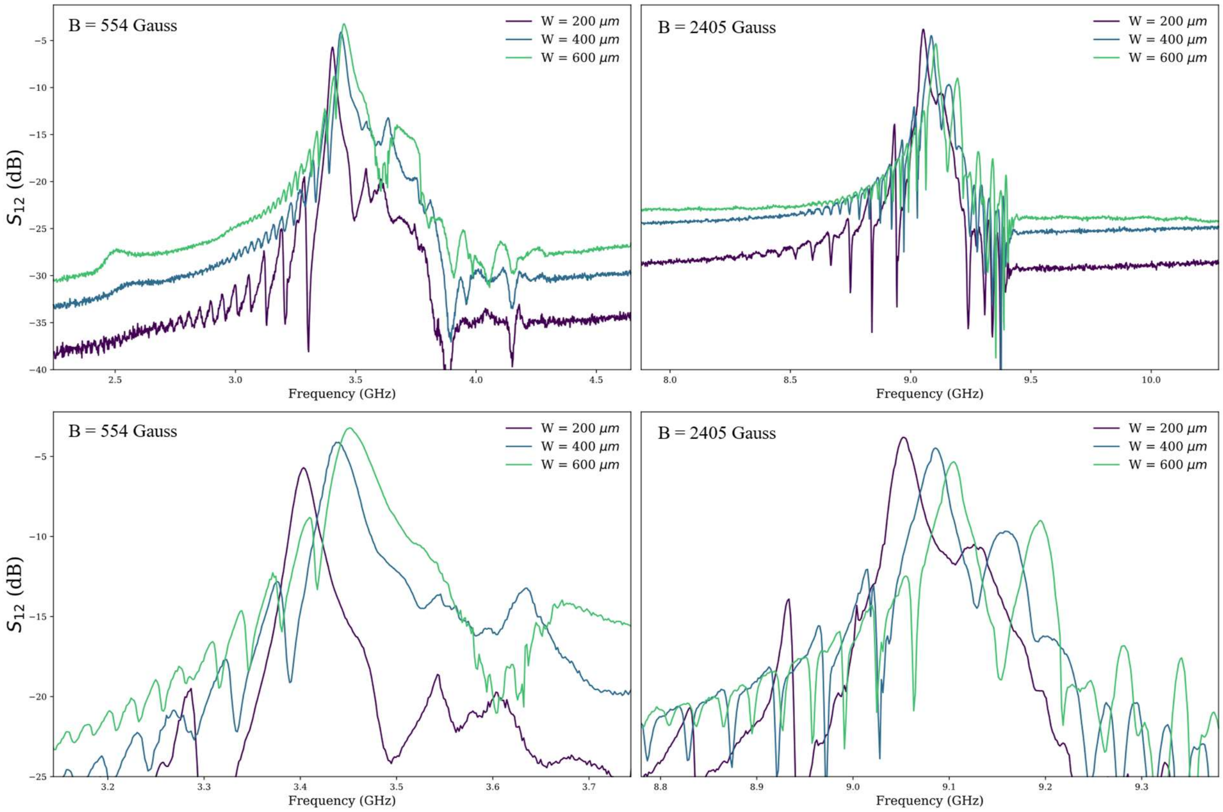

Supplementary Figure 15. S_{12} frequency response of the MSWF at different magnetic flux density (B). The impact of YIG cavity width on the frequency response with constant length of 70 μm.

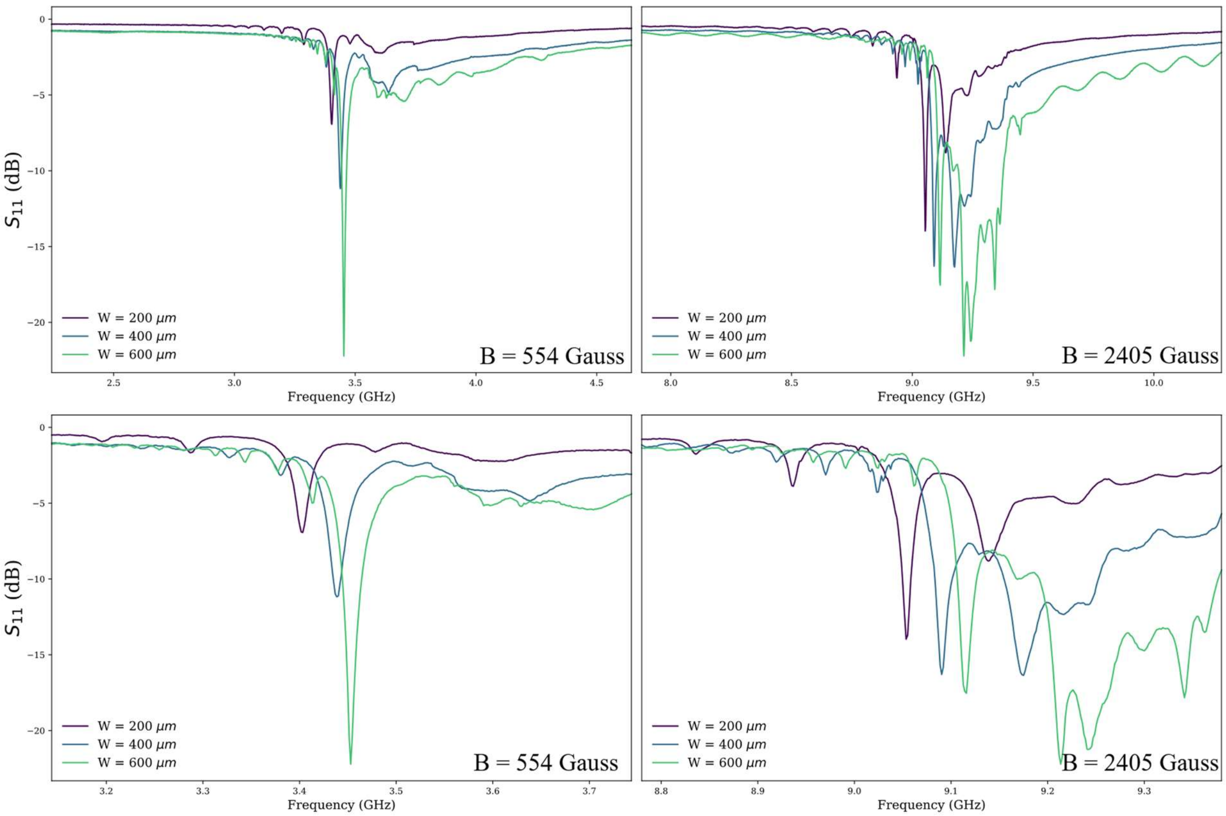

Supplementary Figure 16. S_{11} frequency response of the MSWF at different magnetic flux density (B). The impact of YIG cavity width on the frequency response with constant length of 70 μm.

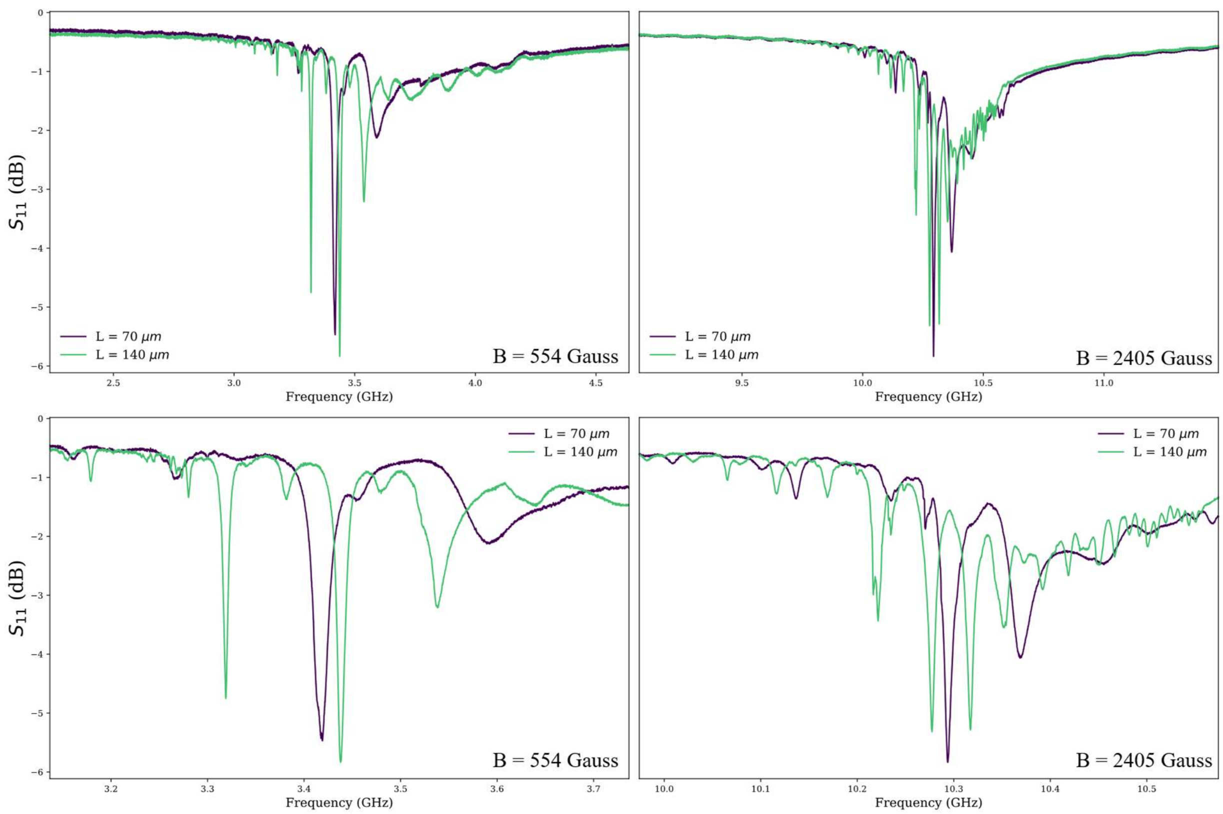

Supplementary Figure 17. S_{11} frequency response of the MSWF at different magnetic flux density (B). The impact of YIG cavity length on the frequency response with constant length of 150 μm.

Supplementary Note 10: Circuit Modeling Procedure for Magnetostatic Wave Filters (MSWF)

Using the prescribed circuit model procedure, it is possible to accurately fit the frequency response of the two-port MSWF. By applying the appropriate circuit parameters and modeling techniques, the circuit model can replicate the observed frequency characteristics of the MSWF. This allows for a better understanding and analysis of the filter's performance and behavior. For the MSWF, the off-resonance Z_{11} impedance is the same as the Z_{series}, and the off-resonance Z_{12} impedance is mainly due to the impedance of the inductor L_c, which can be calculated by the imaginary part of the off-resonance Z_{12}. Thus, the L_s' in MSWF can be expressed as:

$$L_s' = L_{s1} - L_c \quad \text{(S12)}$$

Supplementary Figure 18 shows the comparison of the Z_{12} and S_{12} measurements and the single mode circuit model. In this single-mode circuit model, only one resonance tank was used. Its parameters can be found in Supplementary Table1. The L_s' and L_c of the circuit is 0.34 nH and 30.64 pH, respectively.

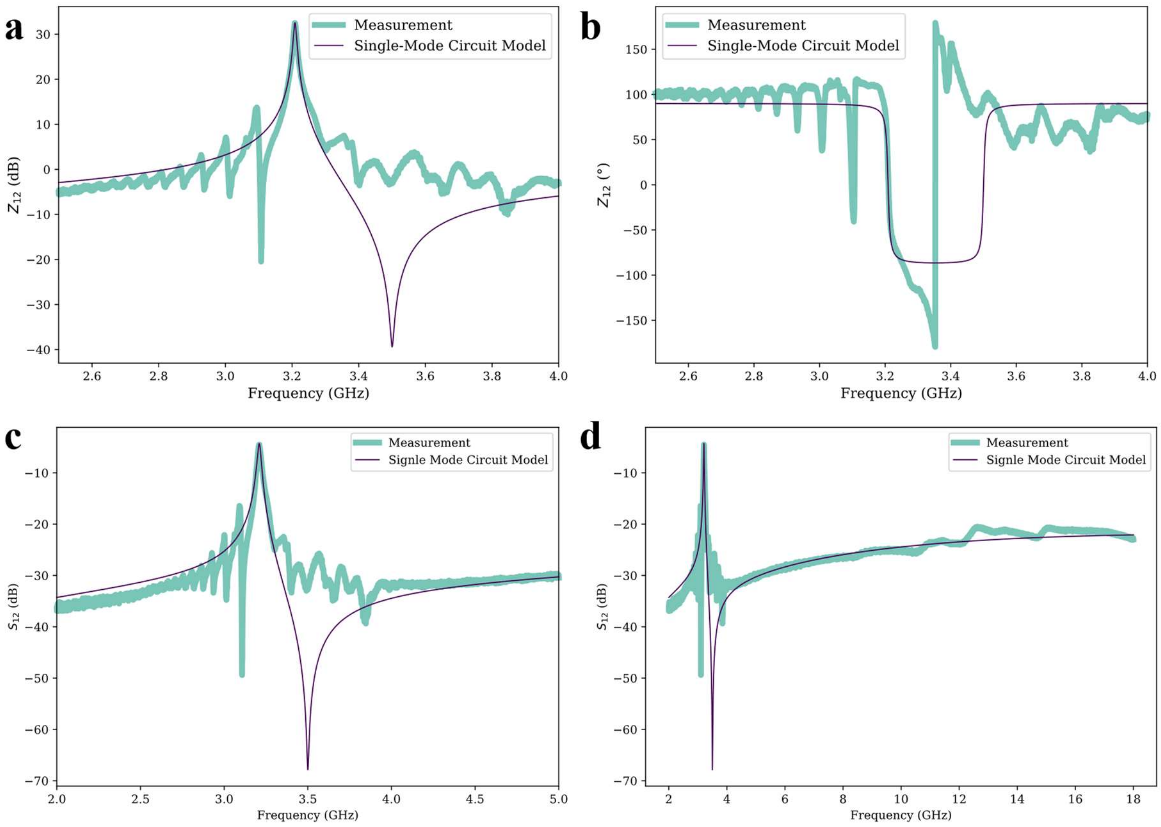

Supplementary Figure 18. Examples of the single-resonance recursive fitting using measurement of MSWF with width of 200 μm and length of 70 μm with transducers width of 4 μm. (a) Magnitude of Z_{12}, (b) Phase of Z_{12}, (c) Magnitude of S_{12} with frequency from 2 to 5 GHz, (d) Magnitude of S_{12} with frequency from 2 to 16 GHz.

Supplementary Note 11: Comparison of S_{12} and S_{21}

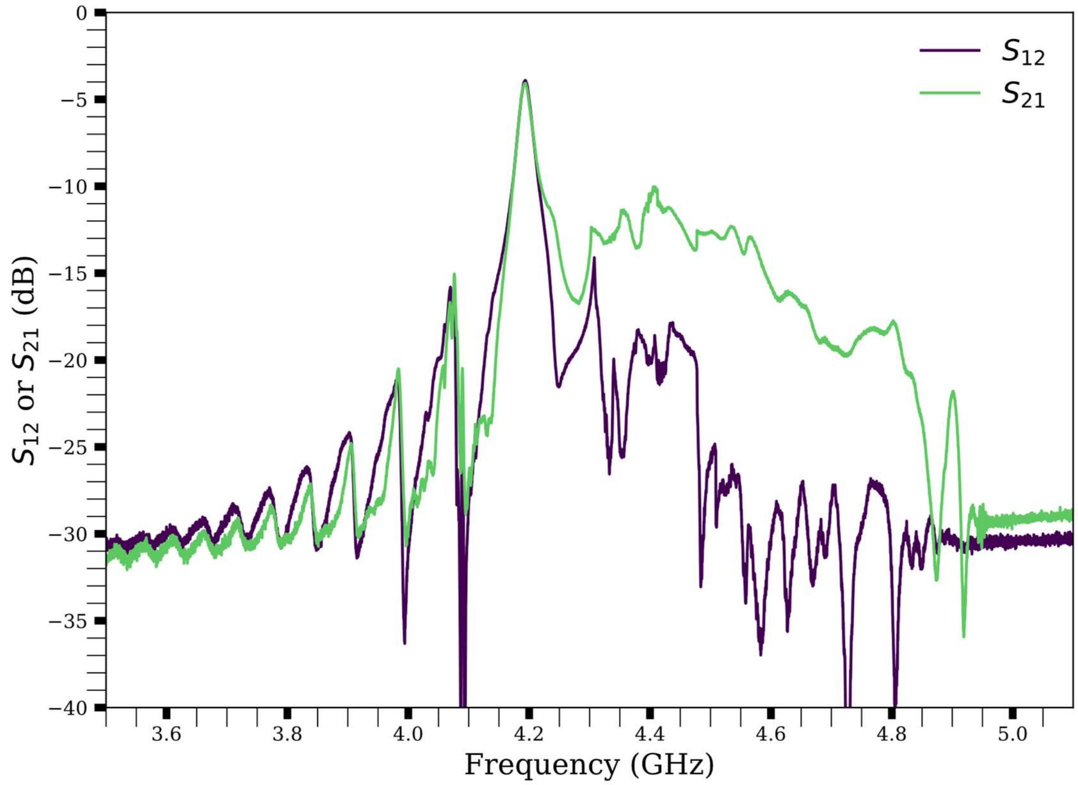

Supplementary Figure 19. Example comparison of the S_{12} and S_{21}. The MSWF with W = 200 µm and L = 70 µm with Al transducer width of 4 µm. The MSWF is biased at a magnetic flux density of 790 Gauss.

Supplementary Note 12: 1 dB Compression Measurement

High power measurements of the fabricated filter were carried out on a MSSW filter with W = 150 μm and L = 70 μm. Supplementary Figure 20 shows the frequency response of S_{12} with different input power. Supplementary Figure 21 reports the input 1dB compression point with respect to different center frequencies.

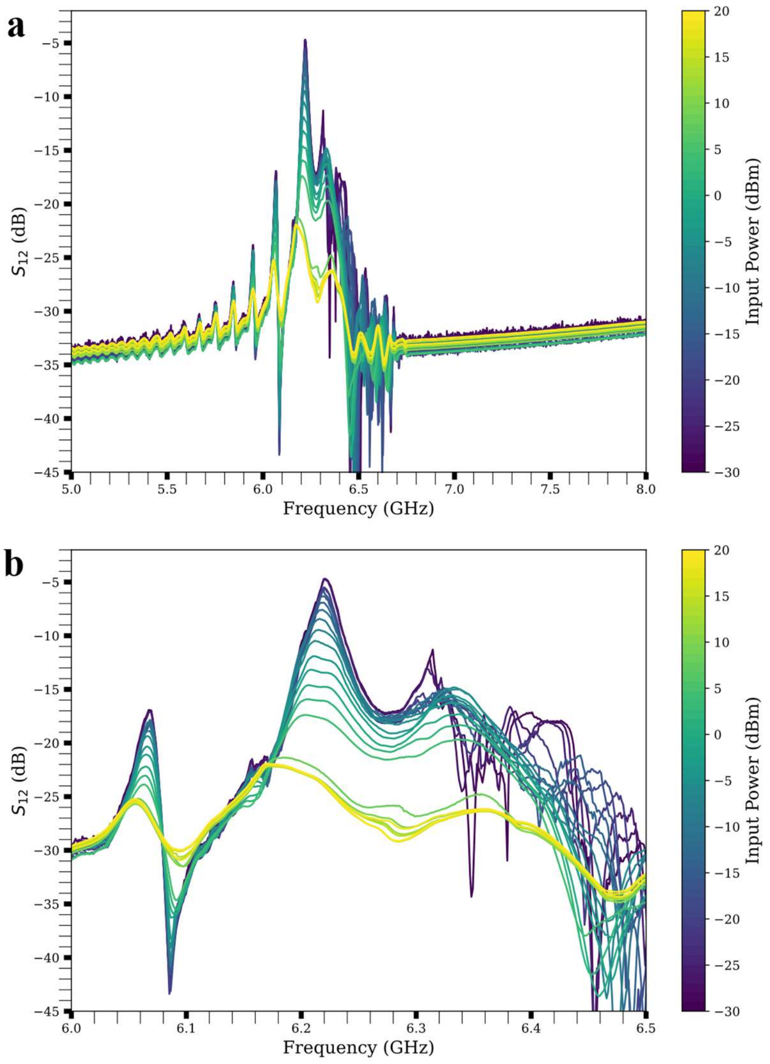

Supplementary Figure 20. Comparison of S12 with different input power by using width = 150 μm, length = 70 μm with transducers on top of the YIG devices. The input power increased from -30 dBm to 20 dBm with a step of 2 dBm.

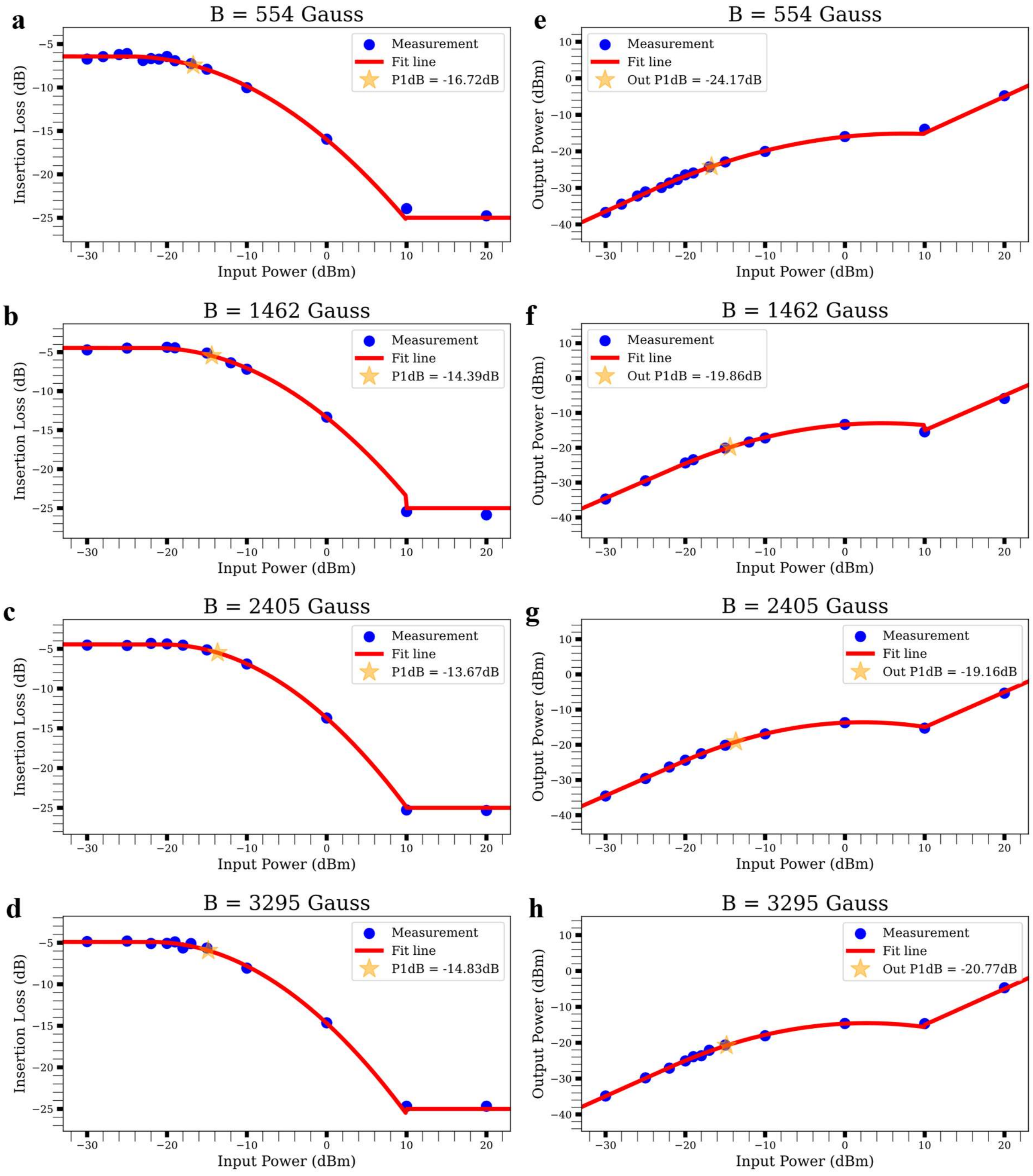

Supplementary Figure 21. Comparison of insertion loss and output power with different input powers for the MSWF with width = 150 μm, length = 70 μm. Effect on insertion loss with (a) frequency of 3.4 GHz, (b) frequency of 6.2 GHz, (c) frequency of 8.9 GHz, (d) frequency of 11.6 GHz. Effect on output power with (e) frequency of 3.4 GHz, (f) frequency of 6.2 GHz, (g) frequency of 10.3 GHz, (h) frequency of 11.6 GHz.

Supplementary Note 13: Intermodulation Intercept Point (IIP3) Measurement

Supplementary Figure 22 shows the schematic for the IIP3 measurement setup. For IIP3 measurement, the two-tone signals (f_1 and f_2) were generated using two signal generators (HP 83712A and Gigatronics 2520A). The signal was amplified by a Minicircuit ZX60-83LN-S+ or Minicircuit ZX60-183-S+ for out-of-band IIP3 measurement. For in-band IIP3 measurement, two 30 dB attenuators (BW-S30W2+) were used. Two bandpass filters (Minicircuit ZBSS-10G-S+ for frequencies above 8 GHz, and ZBSS-6G-S+ for frequencies below 8 GHz) and two circulators (DiTom Microwave DMC6018 for frequencies above 6 GHz, and DiTom Microwave D3C2060 for frequencies below 6 GHz) were utilized to clean the signal from the amplifiers. After that, two signals were combined in a power combiner (Krytar 6020265) and connected to another circulator and finally to the DUT (the MSWF device under test). The output of the YIG filter device was connected to another circulator and then to the spectrum analyzer (HP 8563E).

SI Figure 23, 24, 25, 26 and 27 show the in-band and out-of-band IIP3 measurements.

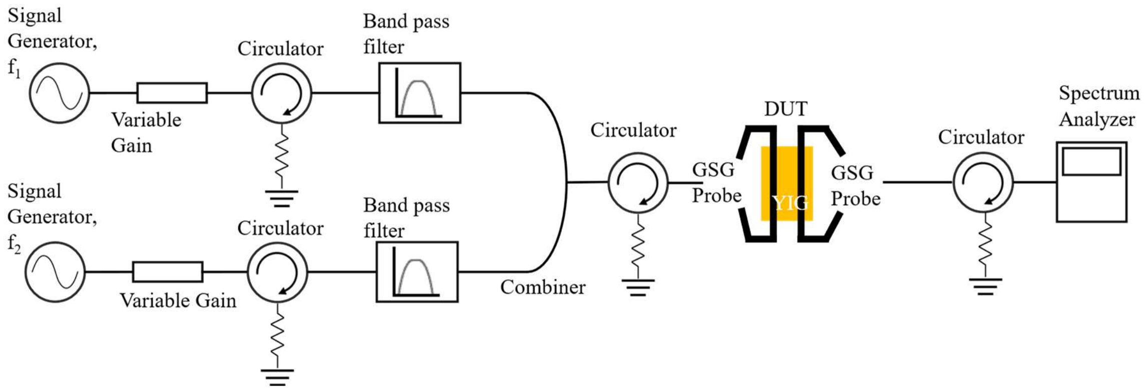

Supplementary Figure 22. IIP3 measurement setup schematic.

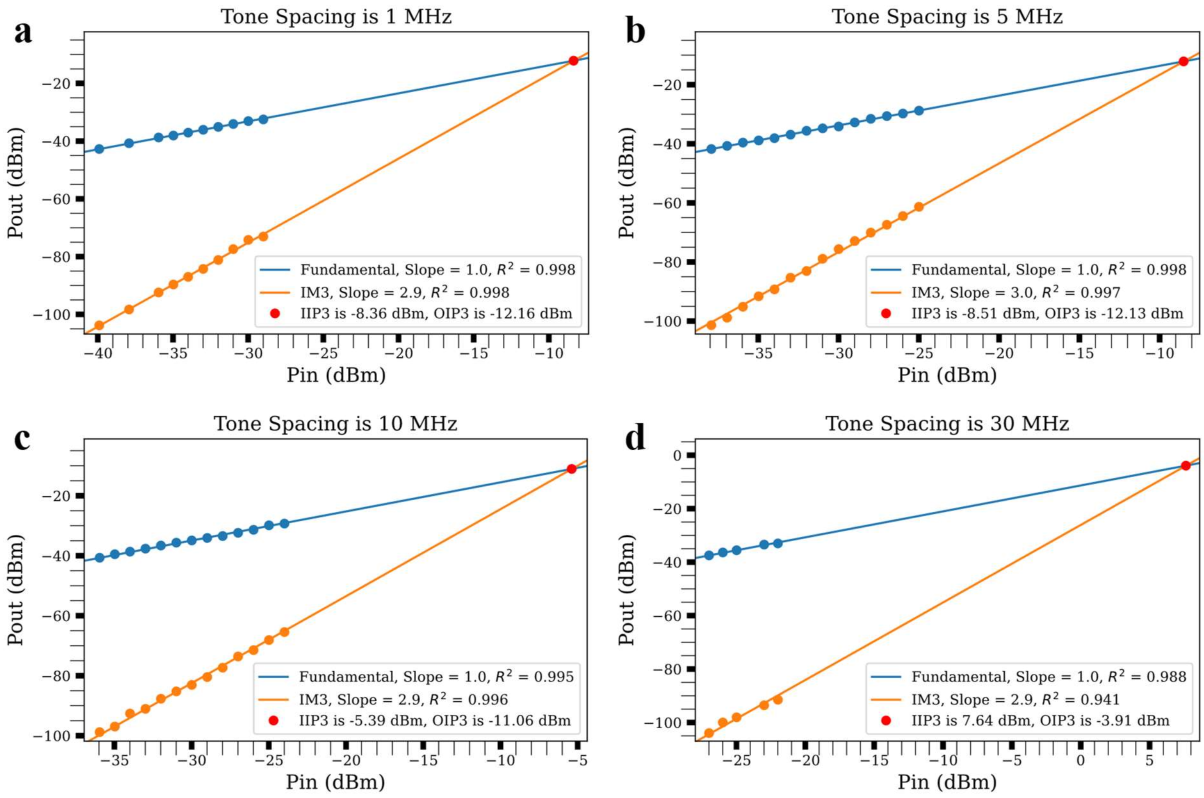

Supplementary Figure 23. In-band third order input intercept point (IIP3). The device is measured at a frequency of 7.6 GHz with tone spacings of (a) 1 MHz, (b) 5 MHz, (c) 10 MHz, and (d) 30 MHz.

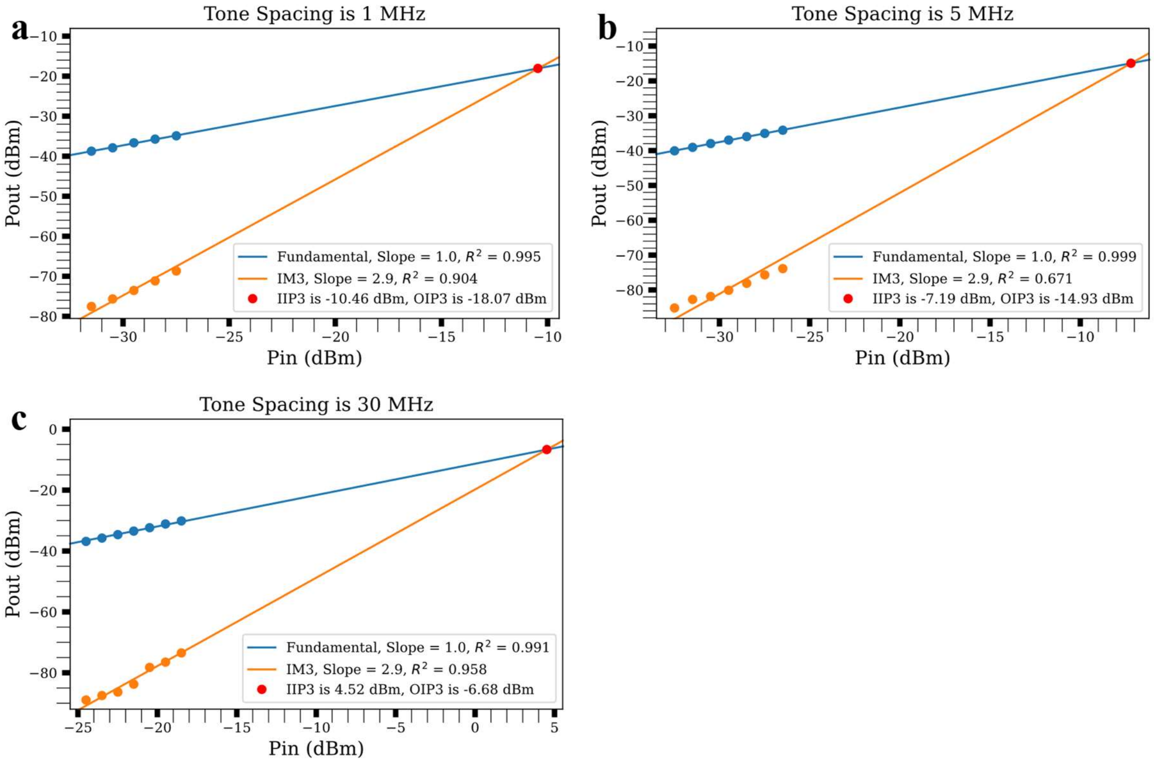

Supplementary Figure 24. In-band third order input intercept point (IIP3). The device is measured at a frequency of 10.1 GHz with tone spacings of (a) 1 MHz, (b) 5 MHz and (c) 30 MHz.

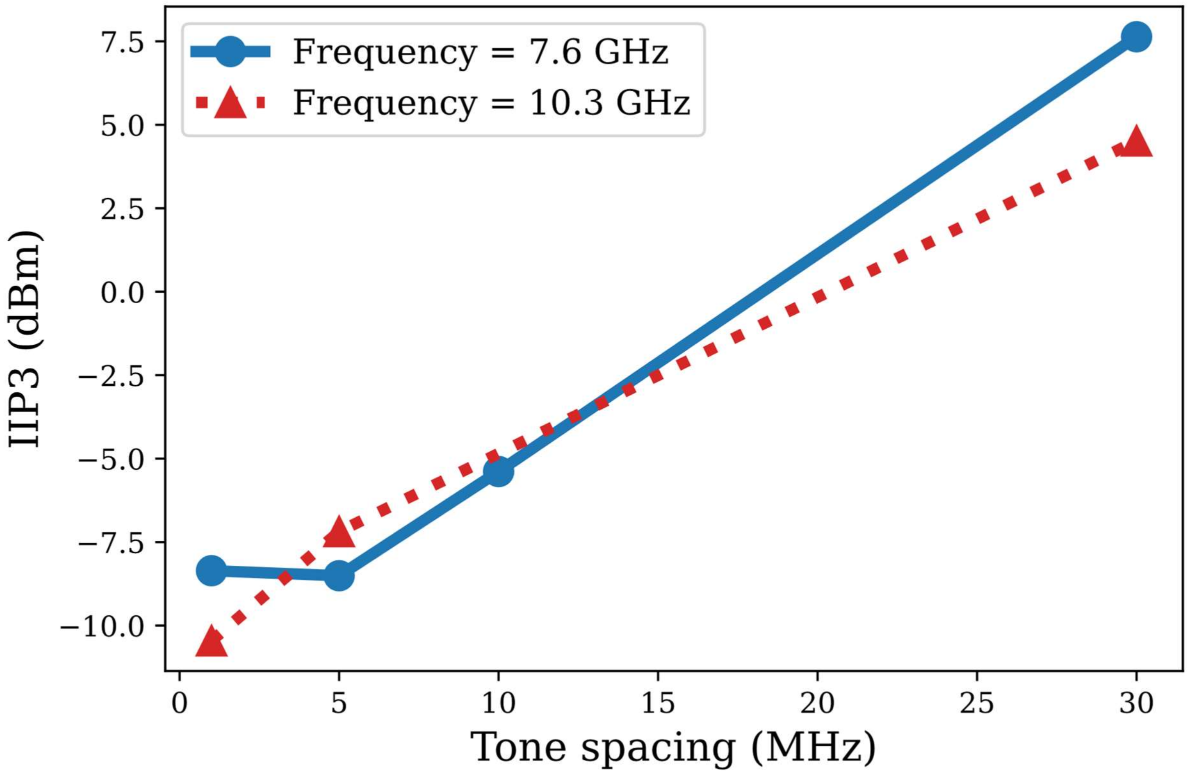

Supplementary Figure 25. A summary of the in-band third order input intercept point (IIP3) measurement result.

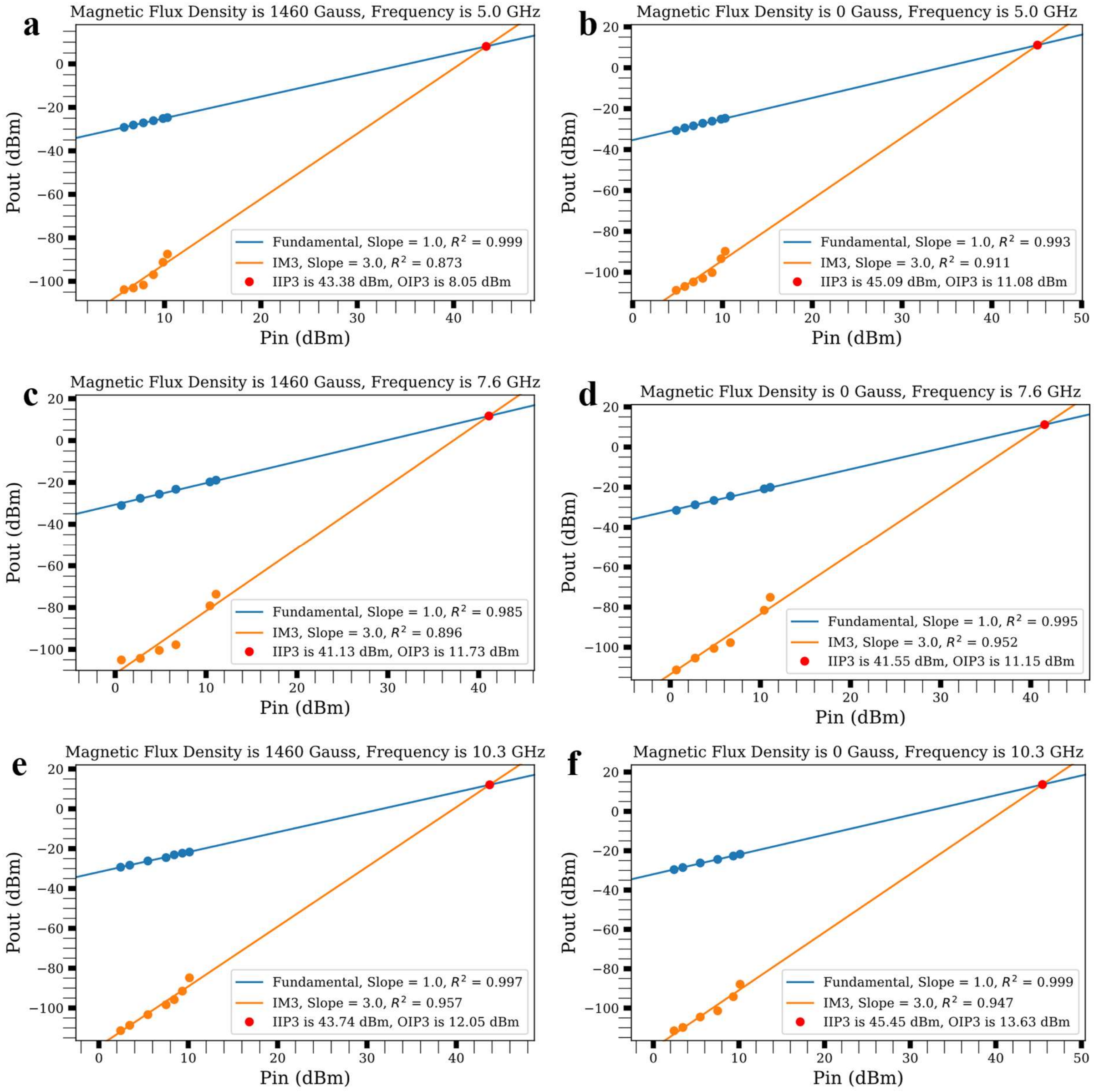

Supplementary Figure 26. Out-of-band (OOB) IIP3 measurement by using width = 150 μm, length = 70 μm devices. The device is measured at frequencies of 5.0 GHz (a) and (b), 7.6 GHz (c) and (d), 10.3 GHz (e) and (f), with an applied magnetic field of 1460 Gauss (a), (c), and (e) and without magnetic field (b), (d), and (f). The tone spacing is 50 MHz.

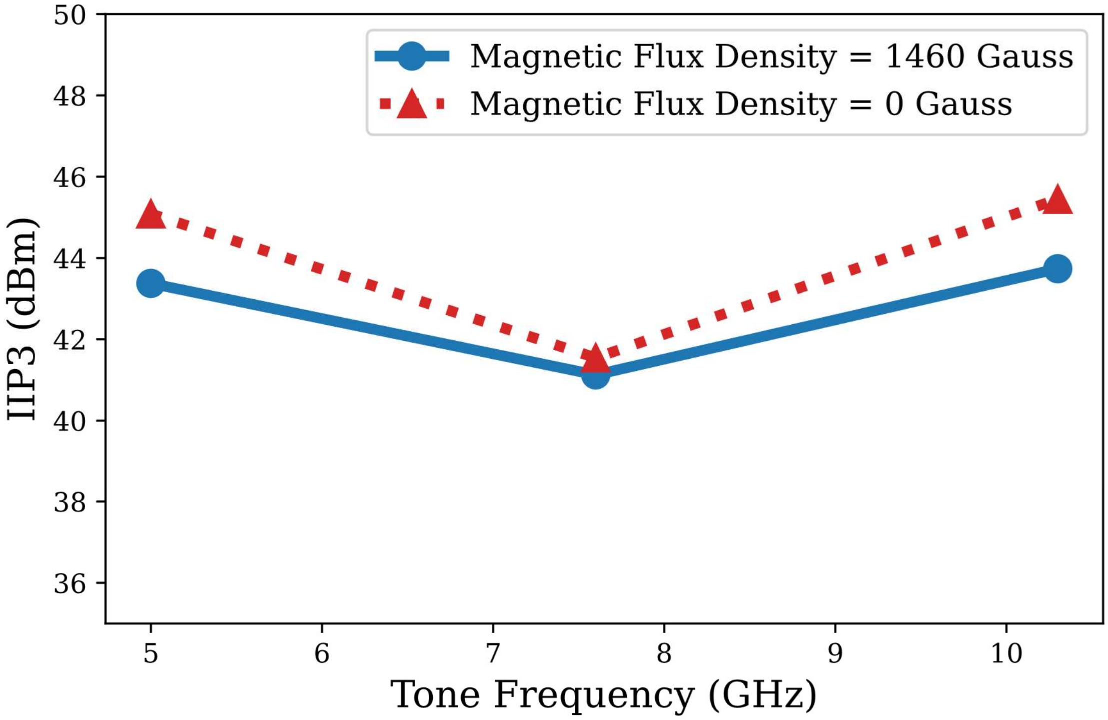

Supplementary Figure 27. Summary of out-of-band (OOB) IIP3 measurement by using width = 150 μm, length = 70 μm devices.

Supplementary Note 14: Magnetic Biasing Circuit Simulation for Magnetic Flux Density

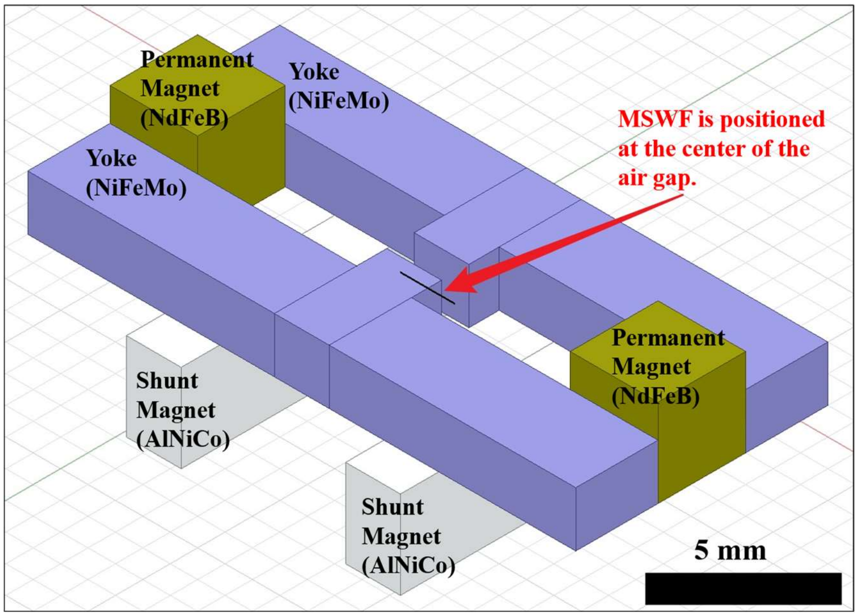

Supplementary Figure 28. Setup of the ANSYS simulation of the magnetic flux density. The two olive boxes are used to represent the NdFeB permanent magnets. The yoke (NiFeMo) is purple in color and has the same shape as the actual device. The two white colored boxes under the yoke are the two shunt AlNiCo magnets.

The coils were excluded from the simulation. This simulation encompassed two scenarios: first, the pair of shunt AlNiCo magnets with full magnetization, and second, AlNiCo magnets with full magnetization but a reversed direction. These scenarios mimic the magnetic bias circuit at its maximum and minimum voltage applications, respectively, and serve as benchmarks for estimating the lowest and highest achievable magnetic fields.

Supplementary Figure 29 shows the magnetic flux density in the YIG chip area when there is a full magnetization of the shunt magnets. The maximum magnetic flux density is 336 mT or 3360 Gauss.

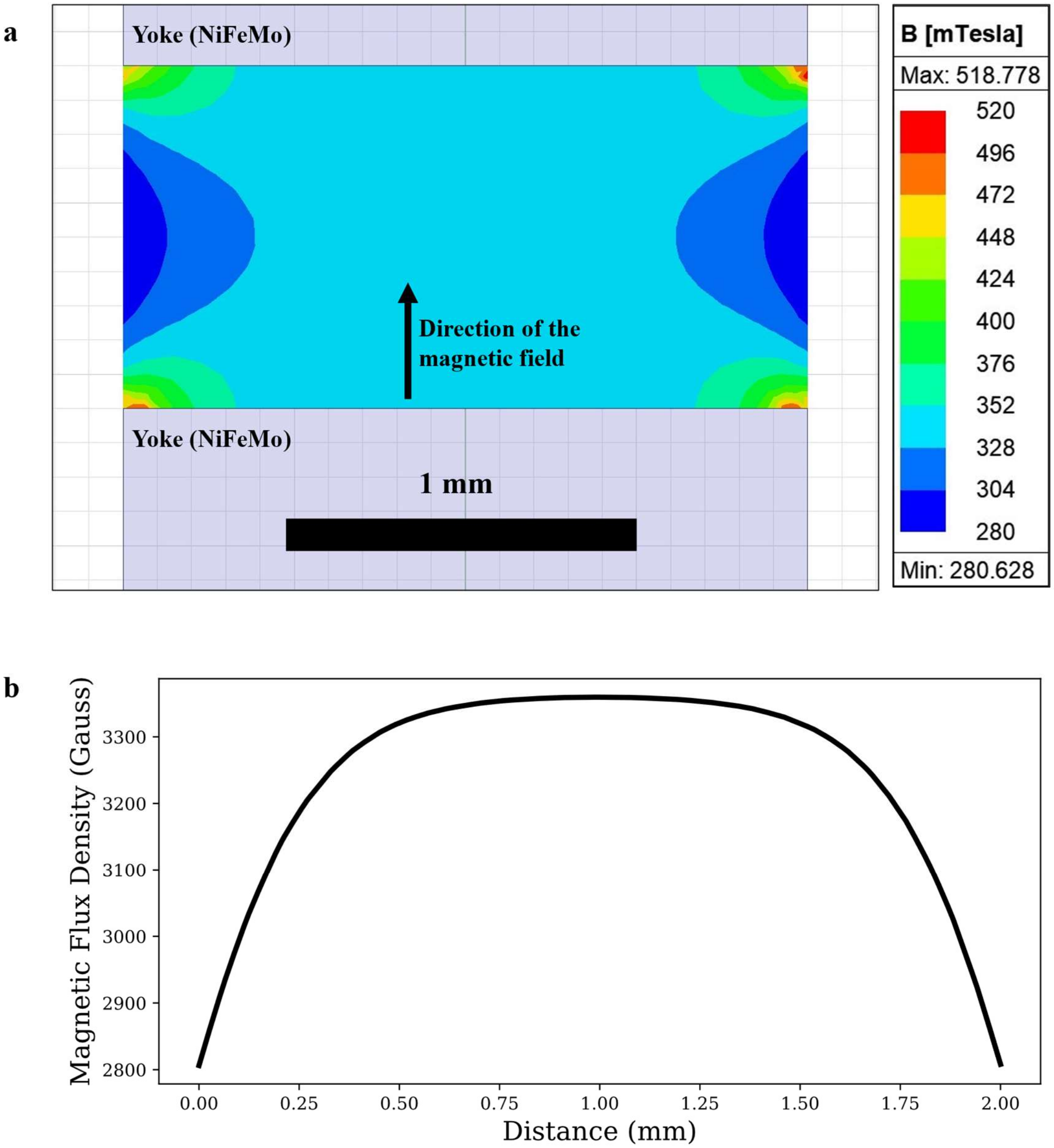

Supplementary Figure 29. (a) Depiction of the simulated magnetic flux density distribution in the central gap region. Two grey boxes, positioned at the top and bottom, symbolize the yokes. This simulation was conducted under conditions of full magnetization of the shunt magnets. (b): Illustration of the simulated magnetic flux density along the center line of the central gap area. Two boxes at the top and bottom represent the yokes. This simulation was performed assuming full magnetization of the shunt magnets. 1 mTesla is equal to 10 Gauss.

Supplementary Figure 30 shows the magnetic flux density in the YIG chip area when there is a reversed full magnetization of the shunt magnets. The maximum magnetic flux density is 45 mT or 450 Gauss.

a

b

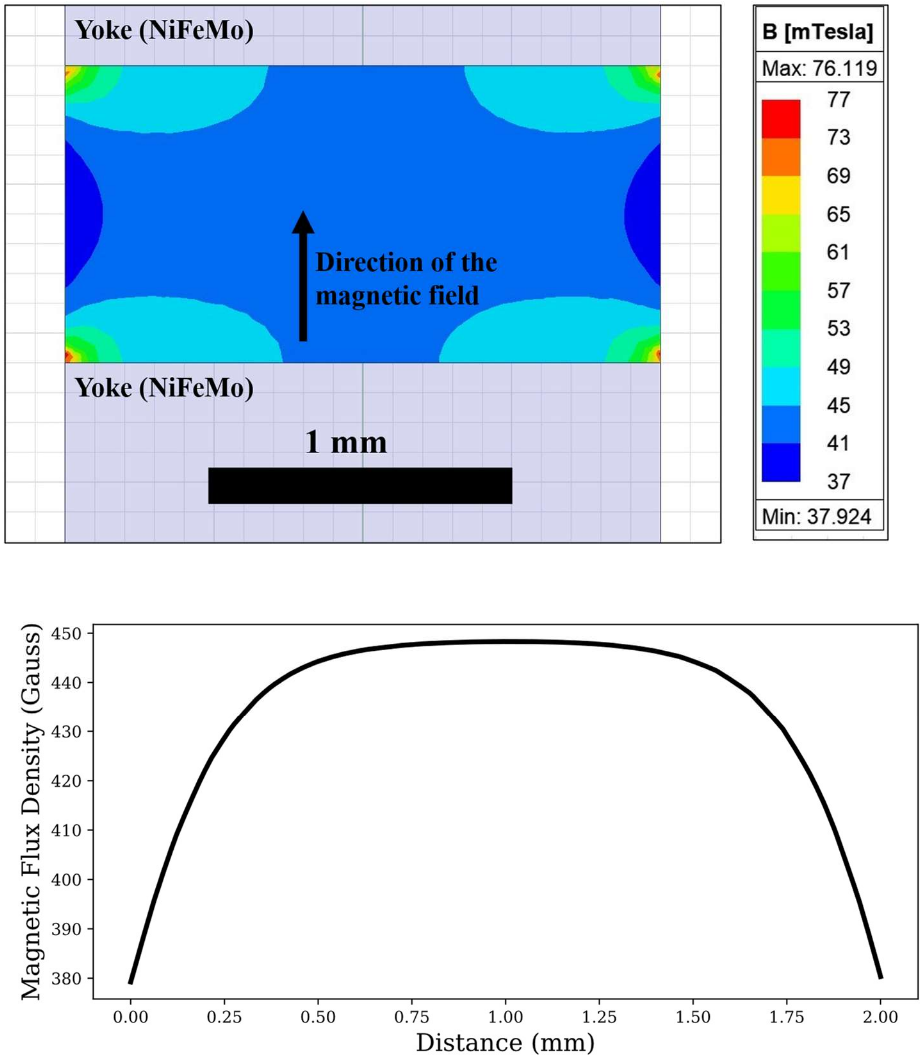

Supplementary Figure 30. (a) Depiction of the simulated magnetic flux density distribution in the central gap region. Two grey boxes, positioned at the top and bottom, symbolize the yokes. This simulation was conducted under conditions of reversed full magnetization of the shunt magnets. (b) Illustration of the simulated magnetic flux density along the center line of the central gap area. Two boxes at the top and bottom represent the yokes. This simulation was performed assuming zero magnetization of the shunt magnets. 1 mTesla is equal to 10 Gauss.

As shown in the main text Figure 6(a), The measured achieved magnetic flux density ranges from 56 mT to 317 mT or 560 to 3170 Gauss. The simulated B- magnetic flux density agrees well with the measured result. The small difference may be due to the air gaps between the shunt magnets and yokes and the air gaps between the permanent magnets and yokes, which narrows the achievable magnetic flux density range. In the simulation, all the contacts were assumed to be perfect. All the magnets and yokes were contacted intimately with no air gaps.

Supplementary Note 15: Magnetic Feld Uniformity of the Magnetic Biasing Circuit

Instead of using a 2 mm thick yoke, a thinner 0.5 mm thick yoke was initially explored to create a separate magnetic biasing circuit. Supplementary Figure 31 shows the measured magnetic flux density as a function vertical position for different capacitor charging voltages. In contrast to the flat flux vs. vertical position observed in Fig. 6 (a), the magnetic flux density profile of the circuit with the 0.5 mm thick yoke exhibits a sharp peak at the center. This indicates that the magnetic biasing circuit is not as uniform as the 2 mm thick yoke. Supplementary Figure 32 shows one example of the filter frequency response where the MSWF was integrated with the magnetic biasing circuit with 0.5 mm thick yoke. In this device, due to the nonuniform magnetic field that is applied to the YIG, the velocity is different in different regions of the MSWF. Thus, return loss reduces and the insertion loss increases. Due to the almost constant bandwidth of the MSSW filter, the requirement of magnetic field uniformity is an absolute constant irrespective of the frequency and the frequency tuning ratio of the MSW device is about 2.9 MHz/Gauss. Thus, it is important for the magnetic bias circuit to provide a high uniformity at all the magnetic bias field levels.

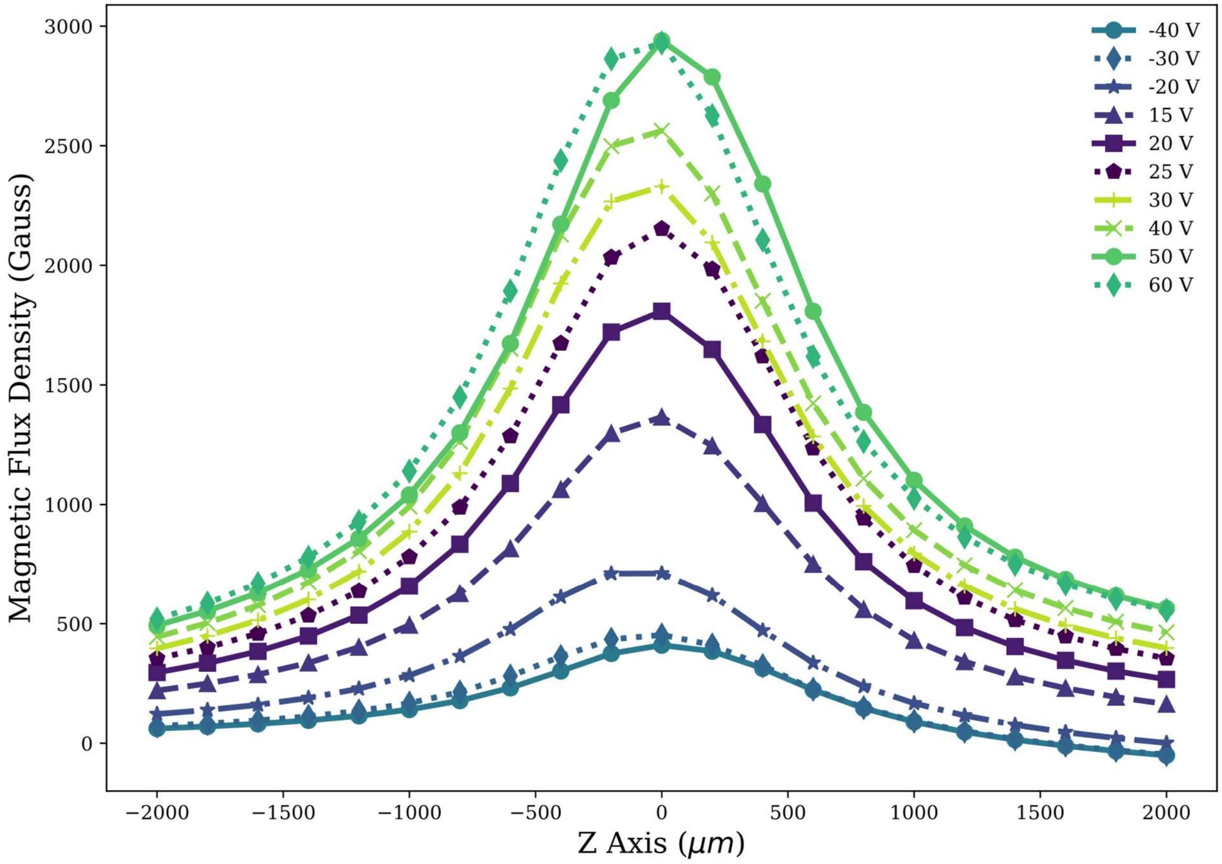

Supplementary Figure 31. Measured magnetic flux density of the magnetic biasing circuit with thin yoke under different capacitor charging voltages.

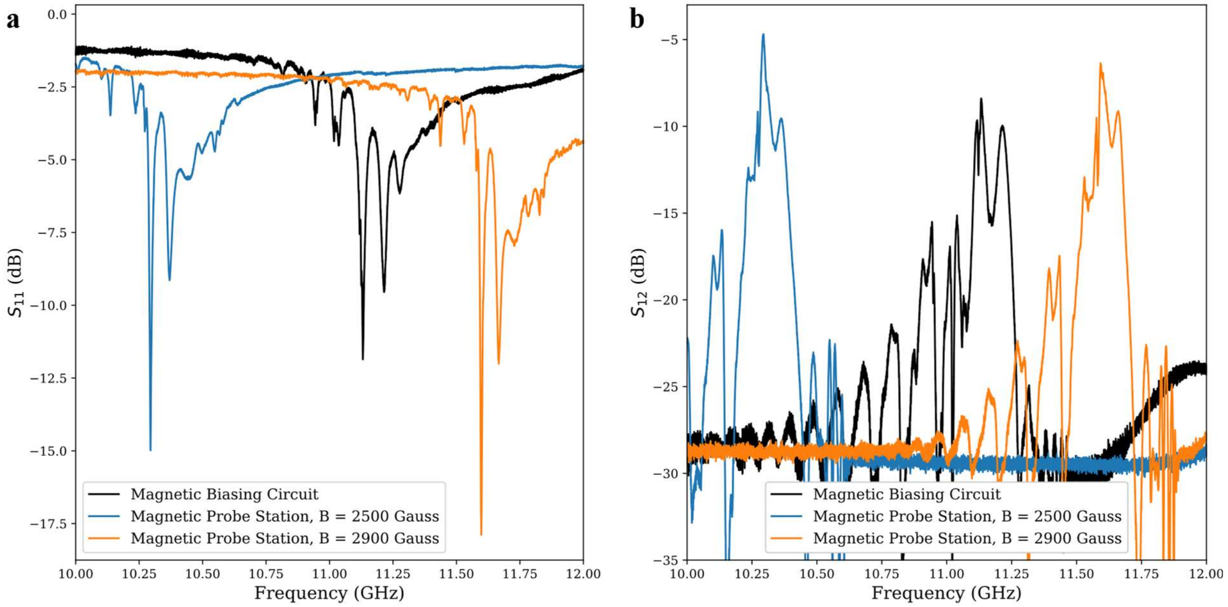

Supplementary Figure 32. Comparison of the frequency response of the MSWF inside the magnetic bias circuit and magnetic probe station where the applied magnetic field of the magnetic bias circuit is nonuniform. (a) S_{11} frequency response, (b) S_{12} frequency response of the width = 150 μm, length = 70 μm MSWF.

Supplementary Figure 33 shows a scenario where the magnetic bias field is uniform, and a similar low insertion loss has been achieved in both magnetic biasing circuit and on the magnetic probe station. The magnetic biasing circuit is biased at a slightly lower magnetic field, which results in the resonance frequency of 9.028 GHz being slightly lower than the resonance frequency inside the magnetic probe station. Overall, the frequency response is similar.

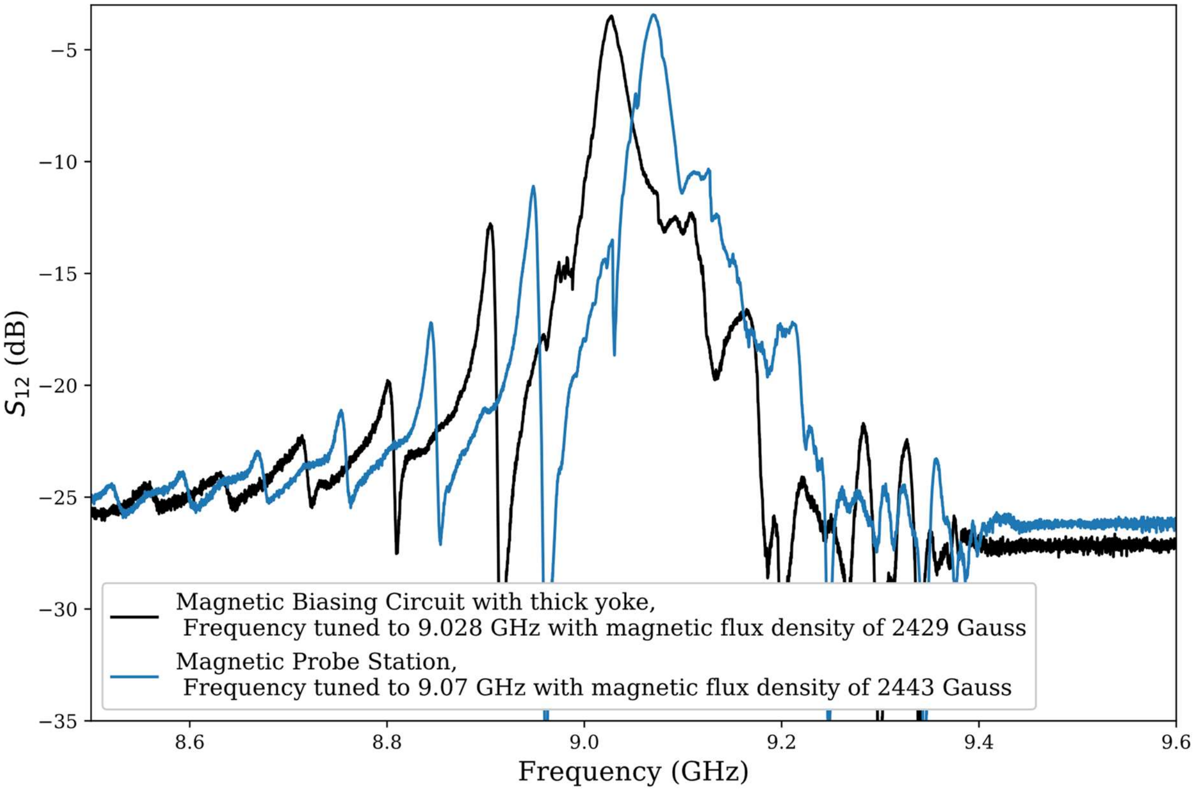

Supplementary Figure 33. Comparison of the frequency response of the MSWF inside the magnetic bias circuit and on the magnetic probe station where the applied magnetic field of the magnetic bias circuit is uniform. S_{12} frequency response of the width = 150 μm, length = 70 μm MSWF. The thick yoke (2 mm thick) is used here for magnetic biasing circuit to achieve better uniformity.

Supplementary Note 16: Magnetically Tunable Notch Filter

Supplementary Figure 34 illustrates an MSWF reconfigured into a band stop filter, where the two ports were connected by aluminum transducers routing on top of the YIG cavity. To minimize inductance and resistance of the line, the aluminum transducers were designed to be as wide as possible in regions not on top of the YIG. Each YIG cavity had a width of 200 μm and a length of 70 μm. In Supplementary Figure 35, the measured S_{12} frequency response of the band stop filter comprising 9 stages of YIG cavities. This filter demonstrates less than 1.5 dB of pass band insertion loss and greater than 20 dB rejection in the tunable notch.

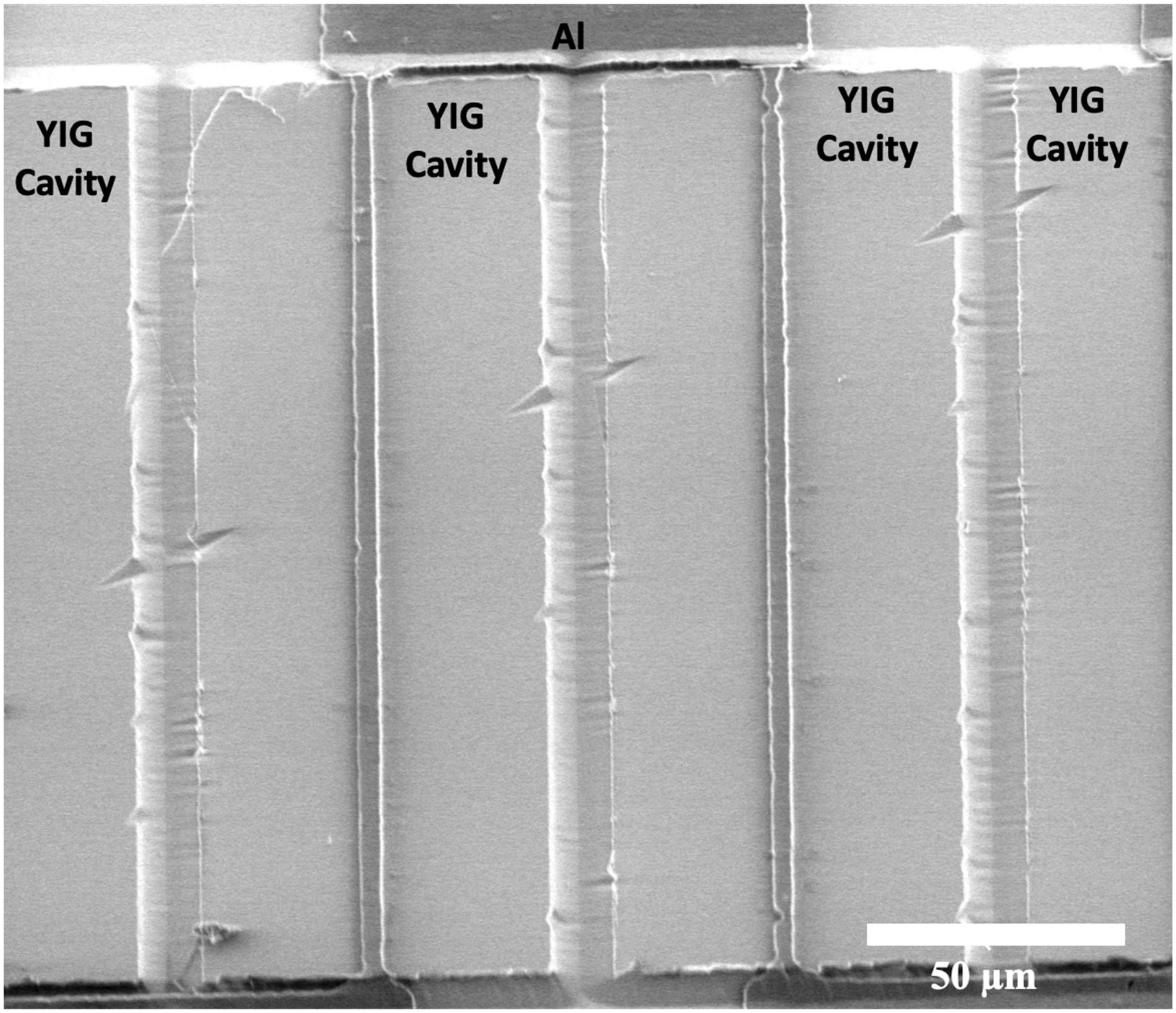

Supplementary Figure 34. SEM image of magnetostatic wave notch filter.

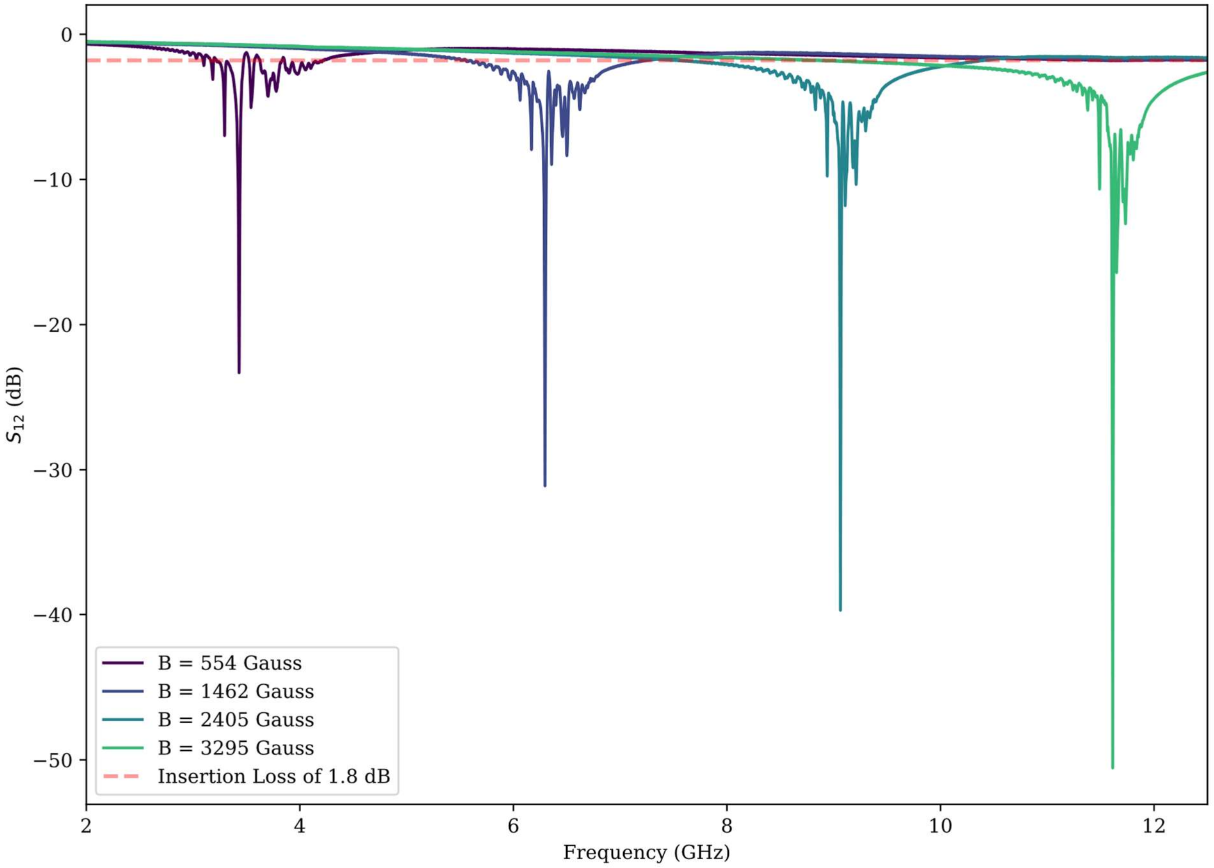

Supplementary Figure 35. Measured S_{12} frequency response of magnetostatic wave notch filter measured with magnetic field supplied by the magnetic probe station.

Reference

1. Castéra JP, Hartemann P. Magnetostatic wave resonators and oscillators. *Circuits, Systems and Signal Processing* **4**, 181-200 (1985).

2. Tsai CS, Qiu G. Wideband Microwave Filters Using Ferromagnetic Resonance Tuning in Flip-Chip YIG-GaAs Layer Structures. *IEEE Transactions on Magnetics* **45**, 656-660 (2009).

3. Wu J, Yang X, Beguhn S, Lou J, Sun NX. Nonreciprocal tunable low-loss bandpass filters with ultra-wideband isolation based on magnetostatic surface wave. *IEEE transactions on microwave theory and techniques* **60**, 3959-3968 (2012).

4. Du S, Yang Q-H, Fan X, Zhang H. High selectivity and compact tunable bandpass filter using YIG material. *Journal of Applied Physics* **133**, (2023).

5. Micro Lambda Wireless Inc. MLFP 4 Stage Filter Data Sheet. *MLFP-42018*.

6. Chiou Y-C, Rebeiz GM. Tunable 1.55-2.1 GHz 4-Pole Elliptic Bandpass Filter With Bandwidth Control and ${> 50}~{\hbox {dB}} $ Rejection for Wireless Systems. *IEEE Transactions on Microwave Theory and Techniques* **61**, 117-124 (2012).

7. MACOM Corporation. MA46 Series Surface Mount GaAs Tuning Varactors 0.75, 1.25, & 1.5 Gamma Hyperabrupt.

8. Wei Z, Yang T, Chi PL, Zhang X, Xu R. A 10.23–15.7-GHz Varactor-Tuned Microstrip Bandpass Filter With Highly Flexible Reconfigurability. *IEEE Transactions on Microwave Theory and Techniques* **69**, 4499-4509 (2021).

9. MACOM Corporation. MAVR-011020-1141 Solderable GaAs Constant Gamma Flip-Chip Varactor Diode

10. Courreges S, Li Y, Zhao Z, Choi K, Hunt A, Papapolymerou J. A Low Loss X-Band Quasi-Elliptic Ferroelectric Tunable Filter. *IEEE Microwave and Wireless Components Letters* **19**, 203-205 (2009).

11. Schuster C*, et al.* Performance analysis of reconfigurable bandpass filters with continuously tunable center frequency and bandwidth. *IEEE Transactions on Microwave Theory and Techniques* **65**, 4572-4583 (2017).

12. Nath J*, et al.* An electronically tunable microstrip bandpass filter using thin-film Barium-Strontium-Titanate (BST) varactors. *IEEE transactions on microwave theory and techniques* **53**, 2707-2712 (2005).

13. Sinanis MD, Adhikari P, Jones TR, Abdelfattah M, Peroulis D. High-Q High Power Tunable Filters Manufactured With Injection Molding Technology. *IEEE Access* **10**, 19643-19653 (2022).

14. Liu X, Katehi LPB, Chappell WJ, Peroulis D. High-Q Tunable Microwave Cavity Resonators and Filters Using SOI-Based RF MEMS Tuners. *Journal of Microelectromechanical Systems* **19**, 774-784 (2010).

15. Lu R, Li MH, Yang Y, Manzaneque T, Gong S. Accurate Extraction of Large Electromechanical Coupling in Piezoelectric MEMS Resonators. *Journal of Microelectromechanical Systems* **28**, 209-218 (2019).

16. COMSOL multiphysics® v. 6.6

17. Brown JA, Barth S, Smyth BP, Iyer AK. Compact Mechanically Tunable Microstrip Bandstop Filter With Constant Absolute Bandwidth Using an Embedded Metamaterial-Based EBG. *IEEE Transactions on Microwave Theory and Techniques* **68**, 4369-4380 (2020).

18. Dai S, Bhave SA, Wang R. Octave-Tunable Magnetostatic Wave YIG Resonators on a Chip. *IEEE Transactions on Ultrasonics, Ferroelectrics, and Frequency Control* **67**, 2454-2460 (2020).

19. Huijer E, Ishak W. MSSW resonators with straight edge reflectors. *IEEE Transactions on Magnetics* **20**, 1232-1234 (1984).

20. O'Keeffe TW, Patterson RW. Magnetostatic surface-wave propagation in finite samples. *Journal of Applied Physics* **49**, 4886-4895 (1978).
